# PVDF-based solid polymer electrolytes for lithium-ion batteries: strategies in composites, blends, dielectric engineering, and machine learning approaches

**DOI:** 10.1039/d5ra02951a

**Published:** 2025-06-18

**Authors:** Khizar Hayat Khan, Abdul Haleem, Sajal Arwish, Afzal Shah, Hazrat Hussain

**Affiliations:** a Department of Chemistry, Quaid-i-Azam University Islamabad Islamabad 45320 Pakistan hazrat.hussain@qau.edu.pk; b Ningbo Institute of Digital Twin, Eastern Institute of Technology Ningbo Zhejiang 315200 China; c Institute of Physical Chemistry, University of Münster Corrensstraße 28/30 Münster 48149 Germany

## Abstract

Solid polymer electrolytes (SPEs) present a viable alternative to organic carbonates typically used as liquid electrolytes in lithium-ion batteries (LIBs). Among various SPEs, poly(vinylidene fluoride) (PVDF)-based SPEs have received significant attention owing to their excellent film forming ability, chemical and thermal stability, mechanical strength, and electrochemical performance. This review focuses on recent innovative strategies in composites, blends, and dielectric engineering to achieve PVDF-based SPEs with enhanced electrochemical performance. It is divided into four primary sections: (1) PVDF-based composite electrolytes, which explores the role of inorganic fillers and nanomaterials in improving ionic conductivity and mechanical properties; (2) PVDF-based blend electrolytes, highlighting the role of polymer blending in optimizing crystallinity, flexibility, and ion transport; (3) dielectric engineering, describing various strategies of manipulating the dielectric properties of PVDF-based SPEs to achieve optimized electrochemical performance; and (4) the emerging role of machine learning (ML) techniques in accelerating the discovery and optimization of SPEs materials by predicting performance and guiding experimental design. Finally, the review concludes with future perspectives and challenges, outlining the potential of PVDF-based SPEs to address current limitations and pave the way for next-generation energy storage applications.

## Abstract 2

This review highlights recent progress in PVDF-based solid polymer electrolytes for Li-ion batteries, focusing on composites, blends, dielectric engineering, and the emerging role of machine learning in optimizing advanced energy storage devices.

## Graphical abstract

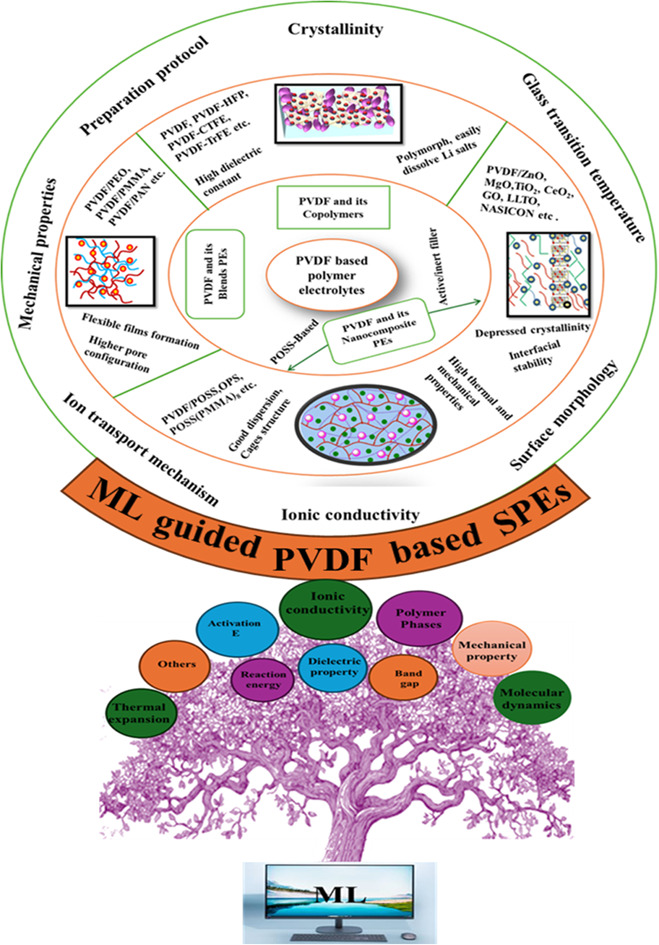

## 1. Introduction

Typically, lithium-ion batteries (LIBs) use a liquid electrolyte composed of a lithium salt dissolved in organic carbonates such as diethyl carbonate (DEC), ethylene carbonate (EC), and others, or their mixtures. The organic solvent must be aprotic to avoid interaction with Li^+^ and exhibit a high dielectric constant that aids in salt dissociation. In addition to organic liquid electrolytes, inorganic liquid electrolytes, such as thionyl chloride with AlCl_3_ and LiCl, are also used in some LIBs, such as LiFePO_4_–graphite.^1–3^ However, liquid electrolytes are associated with serious safety concerns.^4–6^

**Fig. 1:**
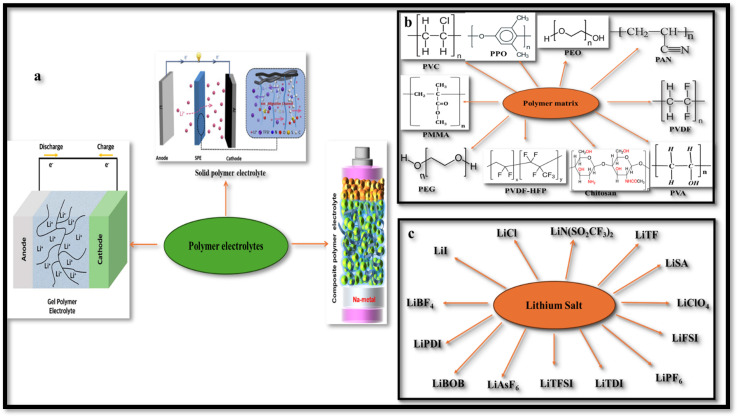
(a) Types of polymer electrolytes. Reproduced with permission from ref. 7. (b) Various types of polymer matrices. (c) Various types of lithium salts.

Solid-state electrolytes (SSEs) (Fig. 1a) represent a viable alternative to organic carbonate-based liquid electrolytes, enhancing stability and safety of LIBs. Solid polymer electrolytes (SPEs) constitute one of the primary classes of SSEs. SPEs have gained prominence owing to their enhanced safety and key attributes such as lightweight design, lower cost, ease of forming thin films into versatile shapes, robust mechanical performance, reliable electrolyte/electrode contact, and greater design flexibility.^8–10^ The choice of polymer hosts for SPEs is mainly dictated by two factors: the presence of functional polar groups with strong electron donor capabilities for coordination with cations, and a minimal hindrance to bond rotation (chain flexibility).^11^ Building on the pioneering work of Wright and Fenton on conductive complexes formed by dissolving alkali salts in poly(ethylene oxide) (PEO),^12^ researchers have explored numerous polymers (Fig. 1b) as potential hosts for SPEs. These include polyimide (PI),^13^ polyvinylidene fluoride (PVDF),^14^ polymethyl methacrylate (PMMA),^15^ polyacrylonitrile (PAN),^16^ and PEO^17^ itself, among others.^18,19^Fig. 1c depicts various types of lithium salts used as the source of lithium ions in SPEs.

**Fig. 2:**
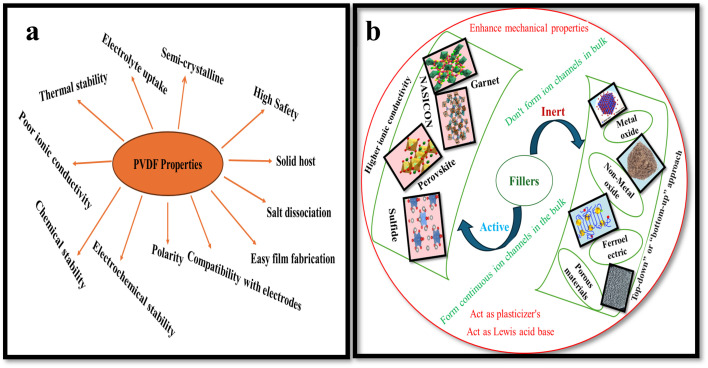
(a) Characteristics of PVDF. (b) Types of filler with examples. Reproduced with permission from ref. 20.

PVDF (Fig. 2a) is regarded as a promising host matrix for SPEs due to its exceptional film-forming ability, robust mechanical strength, high polarity (promoting Li^+^ salt dissociation), strong electrolyte compatibility, and broad electrochemical stability (>4.5 V *vs.* Li/Li^+^).^13–15,17^ However, the semicrystalline nature of PVDF introduces crystalline domains that impede lithium-ion migration, significantly reducing its ion conducting capability.^21,22^ To address this, various strategies have been adopted; including dispersion of ceramic nano fillers (Fig. 2b), including Al_2_O_3_,^23^ CeO_2_,^24^ MgO,^25^ SiO_2_,^26^ and SnO_2_;^27^ blending with other polymers;^28,29^ and manipulating dielectric properties.^30–32^

In recent times, machine learning (ML) has emerged as a powerful tool in materials science, enabling faster, more efficient design and optimization of functional materials.^33,34^ Despite significant progress in fields such as chemistry, biology, and pharmaceuticals, the use of ML in polymer material design, particularly for the development of SPEs, remains relatively underexplored.^35–37^ By analyzing large datasets and applying predictive algorithms, ML can accelerate identification of optimal polymer compositions, filler selection, and processing parameters.^38,39^ Techniques such as random forests, support vector machines, artificial neural networks, and Gaussian process regression have shown promise in predicting key properties, such as ionic conductivity, glass transition temperature, electrochemical stability, and mechanical moduli-based on structural and compositional features.^40,41^ These data-driven approaches significantly reduce experimental workload while expanding the design space for next-generation energy materials.^42^

**Fig. 3:**
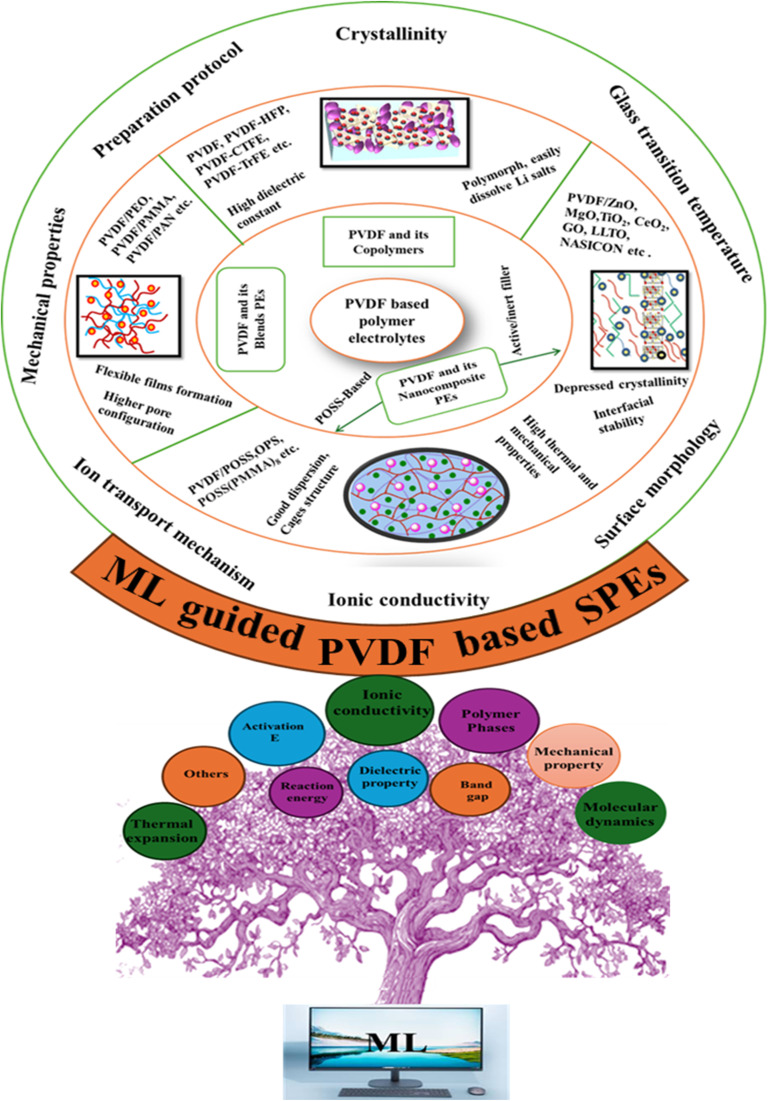
Schematic overview of the PVDF-based solid polymer electrolytes guided by ML.

This review highlights recent advancements in poly(vinylidene fluoride) (PVDF)-based SPEs, with a particular emphasis on PVDF-based composite and blend systems (Fig. 3). A distinctive feature of this work is the comprehensive integration of dielectric engineering principles with composite formulation strategies and polymer blending techniques, presenting a unified perspective on how these elements influence ionic conductivity and electrochemical performance in LIBs. Unlike earlier reviews that have treated these aspects in isolation, we provide a detailed account of the manipulation of PVDF's dielectric properties through filler incorporation, crystallinity modulation, and polymer compatibility aimed at enhancing salt dissociation and ion transport. In addition, we have included a forward-looking section on ML-guided SPEs discovery, introducing recent progress in data-driven materials screening, predictive modeling, and neural network architectures tailored for polymer systems. By integrating experimental design strategies with computational and ML-guided innovations, this review offers a novel interdisciplinary outlook to guide future research and materials optimization in PVDF-based SPEs.

### 1.1. Historical perspectives

**Fig. 4:**
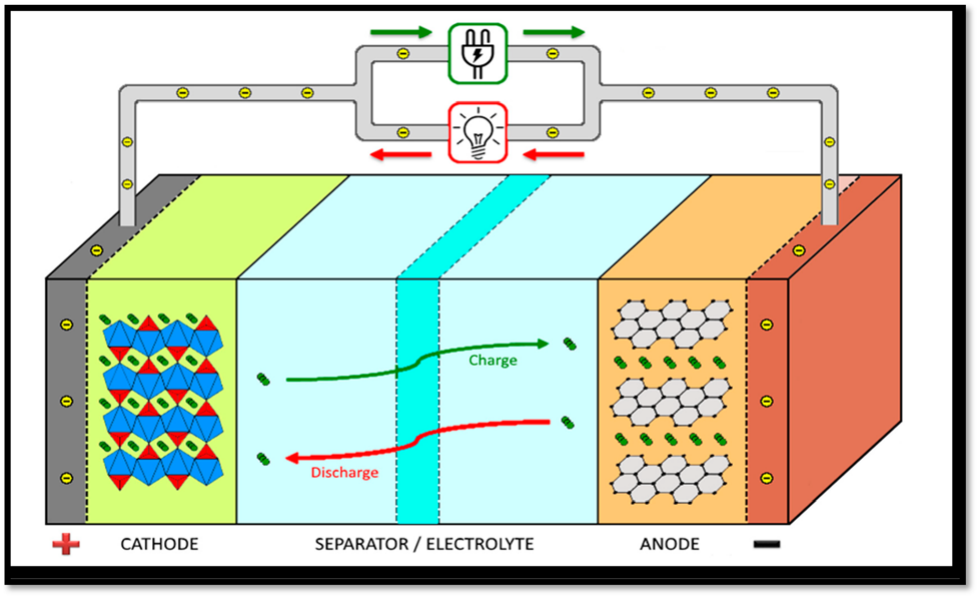
Schematic illustration of the core mechanism of a typical LIB. Reproduced with permission from ref. 48.

The first rechargeable lithium battery, using a lithium-metal anode and titanium disulfide (TiS_2_) cathode, was developed by Stanley Whittingham and his team at Exxon in the early 1970s.^43^ This work laid the foundation for modern LIBs. Whittingham's battery design faced significant safety challenges, including short circuits and fires, largely due to the unstable nature of lithium metal and the moisture sensitivity of TiS_2_. These challenges highlighted the need for further innovation. In 1979, lithium cobalt oxide (LiCoO_2_) was shown to be a viable cathode material for rechargeable lithium batteries by John B. Goodenough, Koichi Mizushima and their coworkers, offering substantially greater energy density than earlier battery technologies.^44^ This material became a cornerstone for modern LIB technology. A crucial breakthrough came in the early 1980s when Yazami, while working with polymer electrolytes, experimentally validated graphite's ability to reversibly intercalate lithium ions – paving the way for its adoption as a standard anode material for rechargeable lithium batteries.^45,46^ Although his work focused on solid electrolytes, it laid the groundwork for the eventual adoption of graphite anodes in liquid electrolyte-based systems, which were commercialized by Sony in 1991. While Yazami's work was foundational, the large scale adoption of graphite anodes in commercial LIBs was enabled by the development of ethylene carbonate (EC)-based liquid electrolytes by Akira Yoshino in the mid-1980s.^47^ Stanley Whittingham, John B. Goodenough, and Akira Yoshino jointly received the Nobel Prize in Chemistry in 2019 for their breakthroughs in the evolution of LIBs, which transformed portable energy storage technology. Fig. 4 schematically illustrates the core mechanism of a typical LIB.

### 1.2. SPEs and ion conduction mechanism

**Fig. 5:**
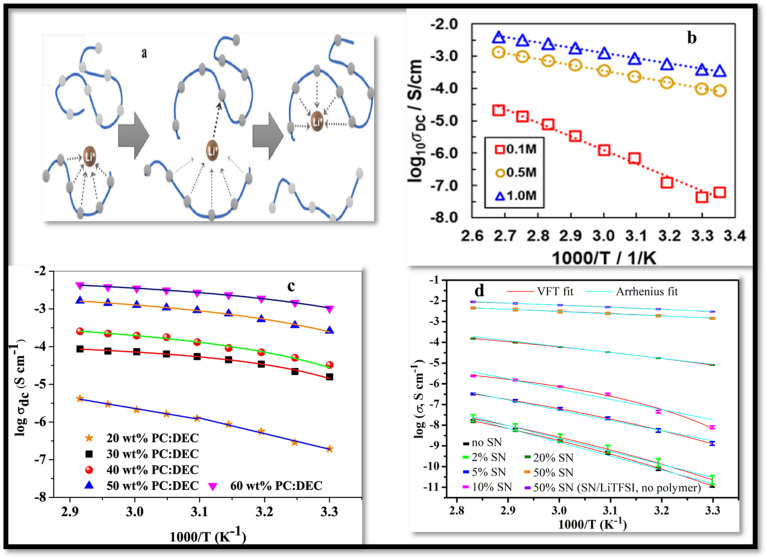
(a) A schematic representation of the Li^+^ ion transport mechanism in a coordinating polymer-based SPE, reproduced with permission from ref. 70. (b) Arrhenius plots of PVDF/Ca(TFSI)_2_ with varying salt concentrations, dried at a constant temperature of 75 °C employing NMP solvent. Reproduced from ref. 71 with permission. (c) Temperature-dependent ionic conductivity of PVDF–HFP : PMMA-LiClO_4_ based gel polymer electrolytes (GPEs) containing varying concentrations of the PC : DEC solvent mixture, reproduced from ref. 65 with permission. (d) A graph illustrates the log *σ versus* 1000/*T* plots for poly ethyl acrylate (EA)-based SPEs containing various concentrations of SN, along with SN/LiTFSI electrolytes without the polymer matrix. The data are fitted using both Arrhenius and VFT models, reproduced with permission from ref. 67.

The study of solid electrolytes began with Michael Faraday's 19th-century observations of ionic conduction in solids, like PbF_2_.^49^ The modern era of SPEs emerged in the 1960s,^50,51^ but it was not until 1973 when Wright and Fenton demonstrated ionic conductivity in PEO/alkali salt complexes.^52^ This breakthrough paved the way for Michel Armand's 1978 proposal of PEO/lithium salt systems as solid electrolytes for rechargeable LIBs, marking a turning point in energy storage technology.^53,54^ In SPE, it is the amorphous phase of the matrix that facilitates ion conduction. In amorphous phase, the disordered polymer chains exhibit segmental mobility above the glass transition temperature (*T*_g_) that facilitates ion mobility.^55–57^ Thus, segmental mobility, chain flexibility, and the degree of amorphousness in the polymer matrix are the critical factors that govern ion transport efficiency.^55^Fig. 5a schematically display the typical ion transport mechanism in SPEs.^58^ Some reports suggest that polymer chains form cylindrical tunnels, facilitating cation coordination through functional groups.^59^ These findings are relevant to the various ion transport models. The Arrhenius model describes the temperature dependent dc conductivity in SPEs as: 
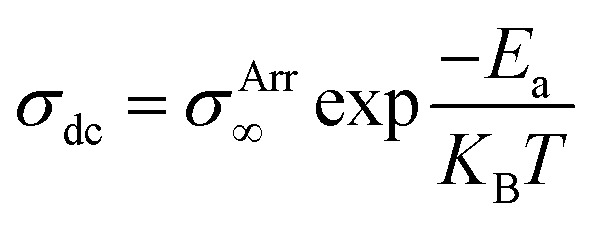
 where *σ*^Arr^_∞_ represents the ionic conductivity at extremely high temperatures. *K*_B_ and *E*_a_ represents, respectively the Boltzmann constant and activation energy.^60^ According to the Arrhenius model, the mechanism of cation transport can be compared to the process of ion conduction in crystals, where ions hop to nearby free sites.^61^ The Arrhenius model describes ionic conductivity as a thermally activated process, exhibiting a linear dependence of log *σ*_dc_ on inverse temperature (1/*T*). The activation energy is derived from the slope of the linear fit of the Arrhenius plot.^17^ In contrast, the Vogel–Tammann–Fulcher (VTF) model emphasizes the association between polymer segmental relaxations and ionic conductivity. The VTF equation, 
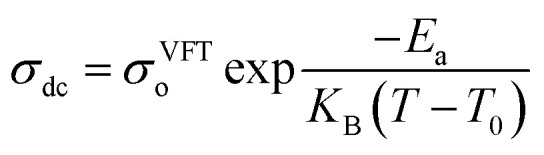
 efficiently captures the non-linear Arrhenius behavior of the temperature dependent conductivity data.^62^ The term “Coupling” is generally used to refer to ion transport being assisted by polymer segmental motion (VTF Model), whereas the term “decoupling” describes ion movement that occurs independently of segmental mobility (Arrhenius Model) such as at low temperatures or in highly crosslinked systems, where alternative transport mechanisms like hopping may dominate. In PVDF-based solid polymer electrolytes, the presence of semi-crystalline regions often restricts polymer chain mobility, particularly at lower temperatures. This reduced flexibility favors ion transport mechanisms that align with the Arrhenius model, where conduction is primarily thermally activated.^14,63,64^ However, when plasticizers, copolymers such as PVDF-HFP, or inorganic fillers are introduced, the crystalline structure becomes disrupted, increasing the amorphous phase content. This enhancement in polymer segmental dynamics supports the use of the VTF model, which better captures the non-linear temperature dependence of ionic conductivity in more flexible systems. Therefore, the applicability of either model is closely tied to the structural features and formulation of the PVDF-based electrolyte. Transitions between Arrhenius- and VTF-type behavior have been observed as the material shifts from a more ordered to a more disordered state, or with increasing temperature.^65–67^ This behavior highlights the necessity of thorough morphological and thermal analysis to accurately determine the most appropriate transport model for a given system. Gohel *et al.*^65^ examined how varying concentrations of the PC : DEC plasticizer mixture affect ionic conductivity (Fig. 5c). For the composition containing 20 wt% PC, the temperature-dependent conductivity followed a linear trend in the log(*σ*) *vs.* 1000/*T* plot, consistent with Arrhenius behavior. This suggests that ion transport at this concentration occurs with minimal involvement of polymer segmental motion. However, at PC : DEC concentrations above 20 wt%, the conductivity plots displayed a distinct curvature, indicating a transition to Vogel–Tammann–Fulcher (VTF) behavior. This shift reflects a strong coupling between ionic mobility and the thermal motion of the polymer chains. The curved nature of the plots implies that, at higher plasticizer levels, ion transport becomes increasingly dependent on the segmental dynamics of the polymer matrix. However, Wang *et al.*^67^ demonstrated that increasing the concentration of succinonitrile (SN) plasticizer in the solid polymer electrolyte (SPE) facilitates the progressive dissociation of Li^+^ ions from the polymer backbone, leading to their preferential coordination with SN molecules (Fig. 5d). This coordination weakens the coupling between ionic transport and polymer segmental motion, thereby triggering a transition from Vogel–Fulcher–Tammann (VFT) behavior to Arrhenius-type ion conduction. These findings elucidate how plasticizers can enhance ionic conductivity by decoupling ion mobility from polymer dynamics, offering valuable insights for the rational design of high-conductivity plasticized SPEs. However, in another study, Shi *et al.*^66^ developed a theoretical model to explain the temperature-dependent conductivity in solid polymer electrolytes by considering melting temperature fluctuations and gelation theory. The study revealed a smooth transition from Arrhenius-type conduction at low temperatures to VTF-like behavior at higher temperatures, consistent with experimental data. This shift occurs sharply at a critical temperature, identified as the polymer's glass transition temperature (*T*_g_), which was found to increase linearly with the melting point. The model provides a clear understanding of how thermal transitions govern ion transport mechanisms in SPEs.

The dielectric constant of the polymer host in SPEs also contributes to the ion conduction efficiency by facilitating salt dissociation that results in higher charge carrier concentration and improved ionic conductivity. This relationship is expressed through the equation 
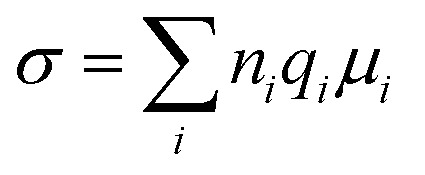
, where “*i*” represents the different types of ions, “*n*” stands for carriers charge concentration, “*q*” signifies the charge of an ion, and “*μ*” designates the mobility of ions.^68^ This suggests that increasing either the charge carriers concentration or the mobility of ionic species will lead to an enhancement in the system ionic conductivity (*σ*). Furthermore, it has been noted that the charge carrier concentration, “*n*,” is primarily influenced by dielectric constant (*ε*_o_) of the host material and dissociation energy (*U*), as specified by equation *n* = *n*_o_ exp(−*U*/*ε*_o_*k*_B_*T*).^69^

### 1.3. PVDF-based composite polymer electrolytes

PVDF and its copolymers, such as PVDF-HFP (polyvinylidene fluoride-*co*-hexafluoropropylene) and PVDF-TrFE (polyvinylidene fluoride-*co*-trifluoroethylene) exhibit versatile properties that can be tailored through the incorporation of nanofillers. These materials are widely studied for their potential in advanced energy storage systems. The addition of various fillers, such as nanoparticles, ceramics, and nanosheets, enhances their mechanical, thermal, and electrochemical properties, making them suitable for applications in batteries, supercapacitors, and flexible electronics (see Table 1 for details).

**Table 1:** Typical Examples of PVDF-based nanocomposites polymer electrolytes

Materials	Fillers	Electrolyte solution	Porosity and uptake%	Conductivity S cm^−1^ and capacity (mA h g^−1^)	References
PVDF	MMT	1 : 1 : 1 (EC : DMC : EMC) with 1 M LiPF_6_	84.08/333	4.20 × 10^−3^ (25 °C)/144	72
PVDF	PMIA	1 : 1 : 1 (DMC : EMC : EC) with 1 M LiPF_6_	−/−	8.1 × 10^−4^/135.29 (0.2C)	73
PVDF	NCC	1 : 1 EC–DMC with 1 M LiPF_6_	−/−	3.73 × 10^−3^ (25 °C)/—	74
PVDF	MV groups	1 : 1 : 1 (EMC : DMC : EC) with 1 M LiPF_6_	67.4/—	1.48 × 10^−3^/136 (0.2C)	75
PVDF	MOF-808	—	−/−	1.58 × 10^−4^ (65 °C)/—	72
PVDF	rGo	1 : 1 (DOL : DME) with 1 M LiTFSI + 0.1 M LiNO_3_	71/380	—/646	76
PVDF	SiO_2_	1 : 1 : 1 (EMC : EC : DMC) with 1 M LiPF_6_	54.1/279.5	—/175.7	77
PVDF	SiO_2_	1 : 1 (EMC–EC) with 1 M LiPF_6_	70/370	2.6 × 10^−3^/132 ©	78
PVDF	SiO_2_	1 : 1 (DEC : EC) with 1 M LiPF_6_	85/646	7.47 × 10^−3^/159 (0.2C)	79
PVDF	SnO_2_	1 : 1 (DMC : EC) with 1 M LiPF_6_	−/−	−/−	80
PVDF	Al_2_O_3_	1 : 1 : 1 (DMC : EC : EMC) with 1 M LiPF_6_	55.8/153.5	2.23 × 10^−3^ (25 °C)/114.2	81
PVDF	Carbon	1 : 1 (DOL : DME) with 1 M LiTFSI + 0.1 M LiNO_3_	−/−	—/827 (0.5C)	82
PVDF	Nano clays/PVP	1 : 1 (DMC : EC) with 1 M LiPF_6_	87.4/553.3	−/−	83
PVDF	DNA-CTMA	(DMC : EC : EMC) with 1 M LiAsF6	−/−	−/−	84
PVDF	Cellulose acetate Al (OH)_3_	1 : 1 : 1 (DMC : EC : EMC) with 1 M LiPF_6_	68.6/403.9	2.85 × 10^−3^/151.97 (C)	85
PVDF	BC	1 : 1 (DEC : EC) with 1 M LiTFSI	−/−	4.2 × 10^−3^ (30 °C)/—	86
PVDF	Al_2_O_3_	1 : 1 (DMC : EC) with 1 M LiFePO_4_	—/230	1.24 × 10^−3^/151.97 (C)	87
PVDF	LiPVAOB	1 : 1 : 1 (EMC : EC : DMC) with 1 M LiPF_6_	—/88.5	2.6 × 10^−4^/120 (0.2C)	88

**Fig. 6:**
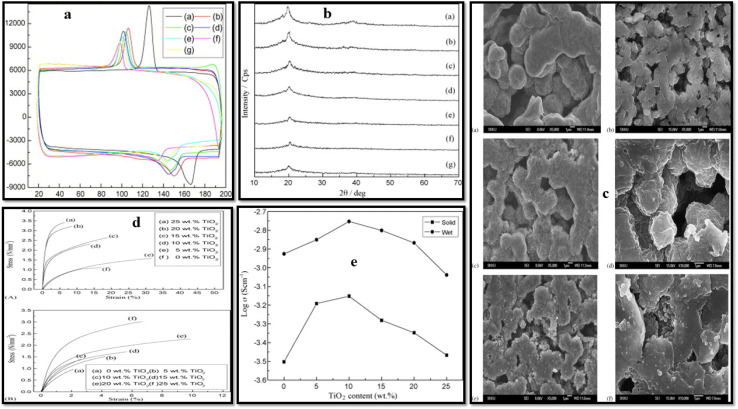
(a) PVDF-based polymer electrolyte membranes DSC traces are as follows: (a) neat PVDF and (b–g) CPEs with increasing TiO_2_ content: 0, 5, 10, 15, 20, and 25 wt%, respectively. (b) XRD profiles of (a) neat PVDF, and (b–g) CPEs with increasing TiO_2_ content: 0, 5, 10, 15, 20, and 25 wt%, respectively. (c) SEM images of CPE with varying filler content: (a) 0, (b) 5 wt% and (c and d) 10 wt% filler content 5000× and 10 000× magnifications, respectively, (e and f) 15 wt% filler content, 5000× and 10 000× magnifications, respectively. (d) Stress *vs.* strain curves of dry (A) and wet (B) CPEs. (e) Ionic conductivity of the CPE with varying filler content.^89^

**Fig. 7:**
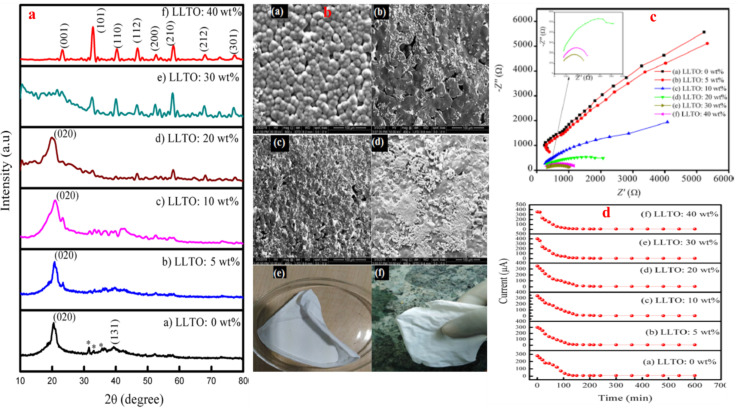
(a) The XRD profiles of PVDF/LLTO CPEs varying filler content. (b) FESEM images of SPE (a) and CPEs with (b) 10, (c) 30, and (d) 40 wt% LLTO. (e and f) Digital images of CPE with 30 wt% LLTO. (c) Nyquist plots of CPEs with: (a) 0 (b) 5 (c) 10 (d) 20 (e) 30 and (f) 40 wt% LLTO. (d) dc current *vs.* dc polarization time for CPEs with varying filler content, reproduced with permission from ref. 90.

Wang *et al.*^89^ designed PVDF-based CPE with TiO_2_ nanoparticles as nanofiller and LiClO_4_ as the lithium salt (Fig. 6). The added nanofiller resulted in deteriorating crystallization of the base polymer as revealed by XRD and DSC analyses (evident from deceasing melting temperature, enthalpy of melting, crystallization temperature, and crystallinity with increasing TiO_2_ content). In addition to thermal properties, the filler also improved mechanical strength compensating for the softening effects of plasticizers like propylene carbonate and ethylene carbonate. The CPE achieved maximum ionic conductivity of 7.1 × 10^−4^ S cm^−1^ for solid dry films and 1.8 × 10^−3^ S cm^−1^ for wet films at 10 wt% TiO_2_, beyond which conductivity declined due to filler aggregation. Sivaraj *et al.*^90^ investigated the effect of active filler, LLTO (Li_0.5_La_0.5_TiO_3_) on the ionic conductivity of PVDF-based CPEs (Fig. 7). The filler profoundly inhibited the crystallization of the PVDF and with 30 wt% LLTO, the crystalline domains of the PVDF almost disappeared as revealed by XRD analysis. FTIR spectroscopy and FESEM confirmed the complexation between PVDF–LiClO_4_ and LLTO, with improved surface morphology and uniform filler distribution. EIS demonstrated that CPEs with 30 wt% LLTO content exhibited the highest dc conductivity of 2.36 × 10^−3^ S cm^−1^ and the lowest *E*_a_ = 0.29 eV. For the optimized CPE, the calculated cation transference number *t*_+_ ≈ 0.853 confirmed that the observed ionic conductivity is predominantly due to lithium-ion transport.

**Fig. 8:**
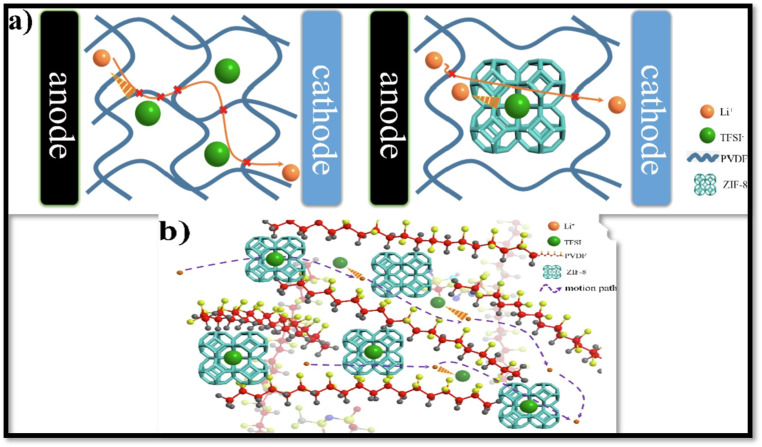
(a and b) The proposed transportation path of Li^+^ through CPEs with ZIF-8 framework-as the filler, reproduced with permission from ref. 92.

ZIF-8 (zeolitic imidazolate framework 8) is a metal organic framework (MOF) displaying highly porous structure, with high adsorption and catalytic performance and thermal stability and has been extensively investigated as photocatalyst for various application including energy systems.^91^ Jiang *et al.*^92^ fabricated the CPE for lithium ionic conductivity by dispersing ZIF-8 in PVDF matrix and were found to exhibit outstanding ionic conductivity of 1.5 × 10^−4^ S cm^−1^ and *t*_+_ = 0.833. Fig. 8 illustrates the likely conduction pathway and state of lithium ions as they migrate through the developed PVDF/ZIF-8 CPE. Within the PVDF structure, ZIF-8 forms a channel that facilitates the migration of lithium ions. The Li^+^ ions are attracted to TFSI^−^ anions within the ZIF-8 pores, enabling migration through ZIF-8. In comparison to their movement through PVDF, the ions experience less resistance when migrating through the ZIF-8 framework. The energy barrier of Li^+^ transportation in ZIF-8 and PVDF was investigated using the Delayed-First Transmission (DFT) method. The energy barrier for Li^+^ transmission through ZIF-8 and PVDF was calculated to be ∼0.07 eV and 0.15 eV, respectively. These findings suggest that Li^+^ preferentially migrates through ZIF-8, indicating that the incorporation of ZIF-8 enhances the ion diffusion efficiency through the CPEs.

Other notable advancement is the incorporation of DNA-CTMA (deoxyribonucleic acid-cetyltrimethylammonium) in PVDF, which enables the development of flexible CPE membranes with excellent mechanical characteristics, such as high elasticity and stretchability.^84^ These properties make DNA-CTMA-modified PVDF membranes ideal for use as foldable separators in flexible energy storage devices. Similarly, the addition of carbon black nanoparticles enhances dielectric properties and mechanical stability, making PVDF-based composites suitable for supercapacitor applications.^93^ ZrO_2_ fillers have been shown to improve membrane porosity, ionic conductivity, and thermal resistance.^94^ The well-connected interstitial gaps created by ZrO_2_ particles facilitate smooth electrolyte absorption, enhancing membrane efficiency.^95^ Graphene oxide nanosheets further improve the mechanical and thermal stability of PVDF-HFP membranes while enhancing electrochemical performance by providing a high surface area for interaction and reducing internal resistance.^96^ Additionally, LLTO (Li_0.5_La_0.5_TiO_3_) nanofillers significantly increases ionic conductivity, enabling high-rate performance and better discharge capacity, which are critical for advanced energy storage applications.^97^ Ceramic powders, such as Al_2_O_3_, reduce crystallinity, acting as plasticizers to improve transport properties and surface compatibility with lithium metal anodes for enhanced cycling characteristics of LIB.^98,99^ Additives such as PVP (polyvinylpyrrolidone) reduce the degree of crystallinity, increasing pore size and improving ion transport pathways.^83^ The solvent section is also very critical in determining the performance of PVDF-based CPEs. For instance, in PVDF/clay-based CPEs, DMAc (dimethylacetamide) has been found to enhance electrolyte uptake and membrane porosity compared to other solvents like DMF (dimethylformamide) or NMP (*N*-methyl-2-pyrrolidone).^83^

#### 1.3.1. PVDF based CPEs with POSS as nanofillers

Polyhedral oligomeric silsesquioxane (POSS) is a unique hybrid nanomaterial with an inorganic silica-like core surrounded by organic functional groups. The fundamental composition of POSS is characterized by the formula (RSiO_1.5_)_*n*_, where R denotes the vertex group, which can comprise hydrogen, alkyl, or some active functional moieties.^100^ POSS exhibits a high degree of symmetry and well-defined molecular geometry, typically featuring a nanoscopic size of 1–3 nm in diameter, inclusive of the vertex groups. The incorporation of POSS nanocages into polymer matrices not only enhances the mechanical strength and thermal stability but also improves the processability of the nanocomposites by reducing viscosity, heat evolution, and flammability.^101–103^ Additionally, POSS possesses many superior properties, such as wearability, oxygen permeability, tenacity, thermal behavior, and mechanical strength.^104–107^

One of the key advantages of POSS is its excellent solubility in many organic solvents. Unlike typical inorganic fillers, which are hard to disperse homogeneously in common organic solvents and often result in agglomeration in PVDF matrix, POSS offers a more viable option for developing composites with uniform dispersion. This characteristic, coupled with its ability to significantly improve mechanical, thermal, and electrochemical properties, highlights the growing appeal of POSS as a nanofiller in advanced nano-structured composite materials for various applications, including its use in LIBs.

**Fig. 9:**
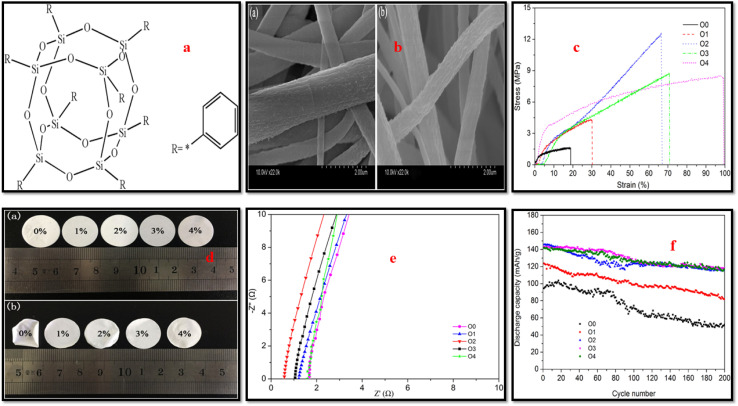
(a) OPS structure, (b) surface characterization of 00 (a) and 02 (b) membranes, (c) stress–strain plots of HPPS membranes, (d) the PVDF/HPPS composite membranes digital images (a) before and (b) after hot treatment, (e) impedance patterns membranes of pure PVDF and HPPS, (f) cycle performance of separators in batteries. Reprinted from ref. 108 with permission.

**Fig. 10:**
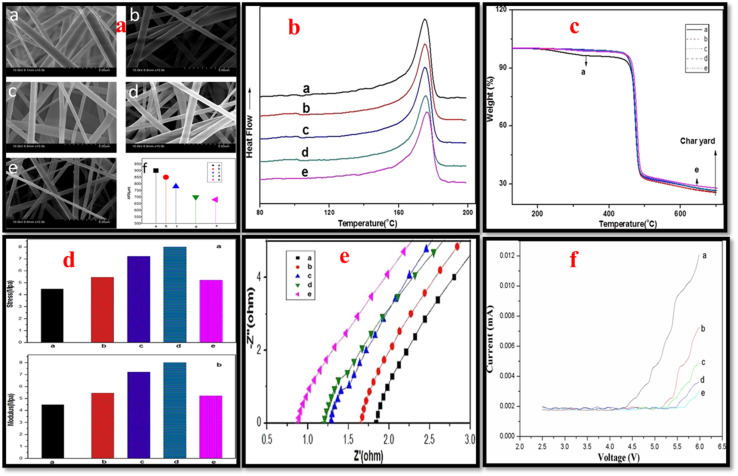
(a) SEM micrographs and AFD, (b) DSC thermograms, (c) TGA curves, (d) Stress and modulus (e) AC impedance plots (f) LSV plots of PVDF/POSS composite membrane with varying POSS content. Here, the POSS content varies from 0, 0.5, 1, 2, to 3 wt% and are represented by a, b, c, d, and e, respectively in the respective figures. Reproduced with permission from ref. 109.

Chen *et al.*^108^ successfully fabricated a novel PVDF/octaphenyl-POSS (OPS) separator, using electrospinning technique (Fig. 9). The organic–inorganic hybrid nature of OPS facilitated homogeneous dispersion in PVDF matrix. The addition of OPS significantly enhanced the tensile strength of the CPE to 12.7 MPa, making it more suitable for transportation and cell assembly. The membrane also exhibited superior thermal stability, showing negligible shrinkage after heat treatment. Electrochemically, the optimized PVDF/OPS membrane (98 : 2 ratio) achieved outstanding ionic conductivity of 4.2 × 10^−3^ S cm^−1^, an expanded stability window to 5.6 V, and a discharge capacity of 145.8 mA h g^−1^. Another similar study by Song *et al.*^109^ reported PVDF/POSS CPE membranes by electrospinning technique (Fig. 10). The CPE membrane exhibited lower crystallinity and thinner fiber diameters compared to pure PVDF fibers. The addition of POSS resulted in enhanced thermal stability as compared with pristine PVDF as confirmed by TGA and improved mechanical performance; increased modulus and tensile strength, suggesting strong interactions between the PVDF matrix and POSS filler. The CPE membrane achieved excellent conductivity of 2.91 × 10^−3^ S cm^−1^ and electrochemical stability window of 5.5 V with 3 wt% POSS, which is attributed to enhanced filler–polymer interactions and Lewis's acid-base coordination. POSS as a nanofiller has certain advantages including the convenient and facile modifications of its reactive R groups attached to its surface. As an example Dapeng *et al.*^110^ prepared POSS-ionic liquid (POSS-IL) and dispersed it as nanofiller in a PEO/PVDF-HFP blend-based CPE. The incorporated POSS-ILs was found to have a significant disrupting effect on the crystallinity of matrix, enhancing its amorphous content that resulted in increased ionic conductivity of 1.5 × 10^−3^ S cm^−1^ at 62 °C and 3.9 × 10^−4^ S cm^−1^ at 22 °C. The CPEs demonstrated reversible capacity recovery and strong cycle performance, highlighting their potential for practical battery applications. In another study, Yi *et al.*^111^ grafted PMMA chains onto the surface of POSS cage constructing a star-like POSS-(PMMA)_8_ hybrid structure and employed it as nanofiller in electro spun PVDF-based CPEs. The modified PVDF matrix exhibited good mechanical strength, thermal stability, and electrochemical properties. The blend demonstrated excellent porosity, elongation, and tensile strength, with the CPEs achieving room temperature ionic conductivity of 4.85 × 10^−3^ S cm^−1^, low interfacial impedance with the Li electrode (256.15 Ω), a wide electrochemical window (6.0 V), and excellent cycle performance.

**Fig. 11:**
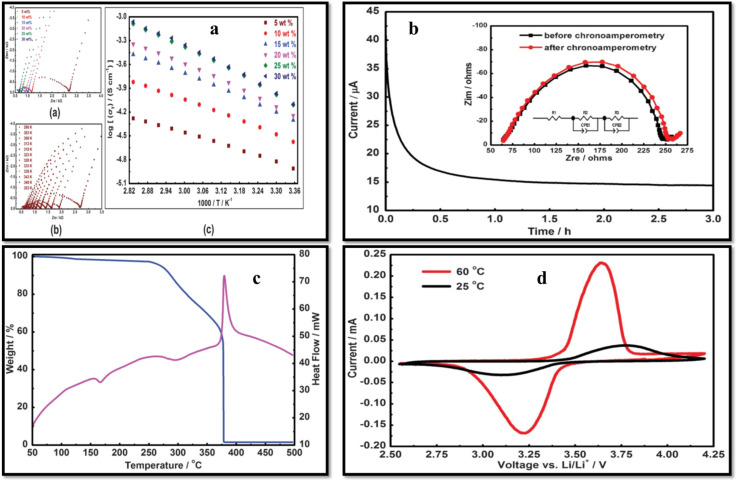
(a) AC plots (a) various concentrations of LiFTSI salt, (b) 5 wt% LiFTSI salt at various temperatures, (c) temperature-dependent ionic conductivity at various salt concentrations. (b) CA and EIS analysis to find the *t*_+_ value (c) DSC and TGA thermograms with 20 wt% LiFTSI, (d) CV of LiFePO_4_/SPE/Li battery at 25 °C (black) and 60 °C (red). Reproduced with permission from ref. 112.

Lin *et al.*^112^ developed an SPE with PVDF/polysiloxane as matrix and LiTFSI as salt. The ionic conductivity increased with salt content, reaching 8.7 × 10^−4^ S cm^−1^ at 80 °C with 30 wt% salt (Fig. 11). The cation transference number (*t*_+_) ranged from 0.249 to 0.478, with the 20 wt% LiTFSI sample exhibiting the lowest interfacial resistance (190 Ω). The electrolyte membrane demonstrated high thermal stability, decomposing above 275 °C, and an electrochemical stability window of 5.17 V at 25 °C. The charge capacity was 144 mA h g^−1^ at 0.2C, with 98% of the discharge capacity retained after 100 cycles, showcasing its potential for high-performance LIBs.

### 1.4. PVDF blends electrolytes for lithium-ion batteries

Another promising strategy for improving the performance of PVDF-based SPEs involves polymer blending. The advancement in PVDF-based polymer blend electrolytes has demonstrated significant progress in addressing challenges such as limited ionic conductivity, poor thermal stability, and low compatibility with lithium-ion electrodes. By optimizing crystallinity, pore structure, and blend composition, blend-based SPE membrane with superior electrochemical performance, mechanical robustness, and safety features can be achieved.^62,113,114^Table 2 summarizes the PVDF blend based SPEs.

**Table 2:** Characteristics of PVDF blends based SPEs

Polymer	Blend polymer	Electrolyte	Conductivity (S cm^−1^)	Capacity (mA h g^−1^)	Porosity%	Uptake%	References
PVDF	PAN	PC/EC with LiClO_4_	—	—	—	—	115
PVDF	PMMA	DMC : EC : EMC (1 : 1 : 1) with 1 M LiPF_6_	—	—	—	—	116
PVDF	NCC	EC : DMC (1 : 1) with 1 M LiPF_6_	—	108 (1C)	—	—	117
PVDF	PEO	DMC : EC (1 : 1) with 1 M LiPF_6_	—	—	—	530	118
PVDF	PEO	DMC : EC with 1 M LiPF_6_	—	—	—	527	119
PVDF	PET	—	—	—	80	270	120
PVDF	PMMA/CA	DMC : EC (1 : 1) with 1 M LiPF_6_	—	—	99.1	323	121
PVDF	PVSK	DOL : DME (1 : 1) with 1 M LiTFSI + 0.25 M LiNO_3_	—	1220	27	—	122
PVDF	P (MMA-*co*-PEGMA	DMC : EC : EMC (1 : 1 : 1) with 1 M LiPF_6_	3.01 × 10^−3^	—	—	372	123
PVDF	PVC	PC with NaClO_4_	1.5 × 10^−4^	—	—	—	124
PVDF	PEGDA-PMMA	EMC : EC : DMC (1 : 1 : 1) with LiCF_3_SO_3_/LiPF_6_	1.0 × 10^−3^ (25 °C)	—	—	—	125
PVDF	PMMA	10 mol% LiClO_4_	3.1 × 10^−5^ (25 °C)	—	—	—	126
PVDF	PE	PC/DEC/EC (5 : 60 : 35) with 1 M LiPF_6_	1.1 × 10^−3^ (25 °C)	—	48	302	127
PVDF	PDMS	EMC : EC : DMC (1 : 1 : 1) with 1 M LiPF_6_	1.2 × 10^−3^	—	55	250	128
PVDF	PI	EC–PC–DEC–VC (35.4 : 17.2 : 45.1 : 2.3) with 1 M LiPF_6_ in	1.3 × 10^−3^	141(C)	—	—	129
PVDF	x-PEGDA	EC–DMC–EMC (1 : 1 : 1) with 1 M LiPF_6_ in	1.38 × 10^−3^ (25 °C)	160.3 (0.2C)	64.6	235.6	130
PVDF	PAN	EC-DMC-EMC (1 : 1 : 1) with 1M LiPF_6_ in	1.45 × 10^−3^	145.71 (0.2C)	—	320	131
PVDF	MC	EMC–DEM–EC (1 : 1 : 1) with 1 M LiPF_6_	1.5 × 10^−3^	110(C)	—	138.6	132
PVDF	PVC	EC : DMC (2 : 1) with 1 M LiPF_6_	1.58 × 10^−3^	125 (0.1C)	62	2 : 30	133
PVDF	PEG/PEGDMA	DEC : EC (1 : 1) with 1 M LiClO_4_	1.70 × 10^−3^	—	71	212	134
PVDF	PLTB	DMC : EC (1 : 1) with 1 M LiPF_6_	1.78 × 10^−3^	138 (0.5C)	70	260	135
PVDF	PVP	BF_4_/PC with 1 M Et_4_N	1.8 × 10^−3^ (25 °C)	—	—	360	136
PVDF	PEGDA-PEO-PPO	PC : EC (1 : 1) with 1 M LiClO_4_	1.9 × 10^−3^	—	32	63	137
PVDF	PMMA	PC : EC (1 : 1) with 1 M LiClO_4_	1.9 × 10 ^−3^	—	—	292	138
PVDF	PANI	DMC : EC (1 : 1) with 1 M LiPF_6_	1.96 × 10^−3^	—	83	270	139
PVDF	PMMA/SiO_2_	—	1.97 × 10^−3^	—	80.1	293.2	140
PVDF	PEO	PC with 1 M LiClO_4_	2.0 × 10^−3^	—	84	210	141
PVDF	TAIC	DEC : EC with 1 M LiPF_6_ and DEC : EC with 1 M TEABF_4_	1.4 × 10^−2^	—	75	—	142
PVDF	HDPE	EC : DMC : DEC with 1 M LiPF_6_ in	2.54 × 10^−3^ (25 °C)	(0.1C)	58	260	143
PVDF	PAN	DEC–DMC–EC (1 : 1 : 1) with 1 M LiPF_6_	2.9 × 10^−3^ (25 °C)		77.7	414.5	144
PVDF	HDPE	—	2.97 × 10^−3^	(140.5C)	71	300	145
PVDF	HTPB-*g*-MPEG	DMC : EC : EMC (1 : 1 : 1) with 1 M LiPF_6_	3.1 × 10^−3^	116(C)	56	350	146
PVDF	PEGDA	DMC : EC (1 : 1) with 1 M LiPF_6_	3.3 × 10^−3^	117 (0.1C)	—	—	147
PVDF	PDPA	PC with 1 M LiClO_4_	3.6 × 10^−3^	—	—	280	148
PVDF	PVC	PC : EC with LiClO_4_	3.7 × 10^−3^	—	—	—	149
PVDF	PEO	PC with 1 M LiTFSI	5.4 × 10^−4^	124(C/5)	44.5	107	150
PVDF	PDMS-*g*-(PPO-PEO)	DMC : EC : EMC (1 : 1 : 1) with 1 M LiPF_6_	4.5 × 10^−3^	120 (1C)	80.1	512	151
PVDF	PBA	DMC : EC : EMC (1 : 1 : 1) with 1 M LiPF_6_	8.1 × 10^−4^ (25 °C)	95 (0.1C)	—	120	152
PVDF	PAN	PC with 1 M LiClO_4_	7.8 × 10^−3^	—	85	300	153
PVDF	PEMA	PC : EC: (1 : 1)	1.5 × 10^−4^ S cm^−1^ (28 °C)	—	—	—	154
PVDF	PMMA	DMC : EC with 1 M LiPF_6_	7.9 × 10^−3^	—	—	260	155

**Fig. 12:**
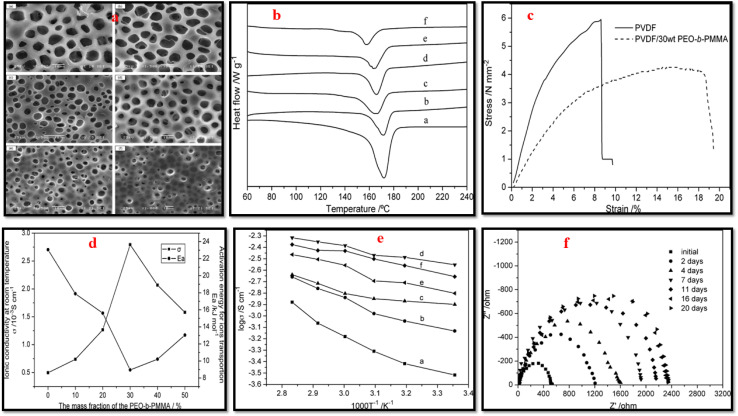
(a) SEM images, (b) DSC curves of PVDF/PEO-*b*-PMMA blend membrane with varying block copolymer content (wt%); ((a) 0, (b) 0.1, (c) 0.2, (d) 0.3, (e) 0.4, (f) 0.5). (c) Stress–strain curves of neat PVDF and blend with 30 wt% block copolymer content. (d) dc-conductivity and activation energy of blend as function of block copolymer content (achieved after soaking in solution of LiClO_4_ and EC–PC (1 mol L^−1^). (e) log *σ vs.* 1/*T* for PVDF/PEO-*b*-PMMA with varying block copolymer content (wt%); ((a) 0, (b) 0.1, (c) 0.2, (d) 0.3, (e) 0.4, (f) 0.5), after soaking in solution of LiClO_4_ and EC-PC. (f) The evolution of the Li/separator/Li cells impedance spectra recorded over time while kept at ambient temperature (reproduced with permission from ref. 157).

Liu *et al.*^29^ fabricated PVDF/PMMA blend membranes using phase inversion method. Electrochemical, physical, and thermodynamic studies confirm the effectiveness of blending PVDF and PMMA for achieving enhanced electrochemical performance. This membrane demonstrated strong compatibility with lithium metal, adequate thermal stability, and a satisfactory ionic conductivity of 2.18 mS cm^−1^ at 26 °C. Moreover, it surpasses the Celgard 2320 (PP/PE/PP) separator both in terms of cycling performance; retaining 130.7 mA h g^−1^ after 200 cycles at 1C and rate capability; 133.3 mA h g^−1^ at 4C. In another study on PVDF/PMMA blend membranes, Yusoff *et al.*^156^ further advanced this approach by developing microporous structure using IL and salicylic acid as a pore-forming agent that facilitated increased electrolyte uptake. The membrane with the optimized composition of 90 wt% PMMA and 10 wt% PVDF exhibited a high *t*_+_ = 0.7922, electrochemical stability up to 4.3 V, and a notable room temperature ionic conductivity of 3.097 mS cm^−1^. Xiao *et al.*^157^ blended PVDF and PEO-*b*-PMMA block copolymer in various compositions and achieved porous membranes using phase inversion method (Fig. 12). The pore density of the membrane improved with increasing block copolymer content, peaking at 30 wt%, which maximized electrolyte uptake to 211%. DSC analysis showed reduced crystallinity and melting temperature with higher PEO-*b*-PMMA content, promoting amorphous regions and enhancing ionic conductivity. The blend with 30 wt% PEO-*b*-PMMA displayed the highest ionic conductivity of 2.79 × 10^−3^ S cm^−1^ compared to 0.49 × 10^−3^ S cm^−1^ for pristine PVDF. In addition, the stress–strain analysis indicated improved elongation properties of blend with a fracture strain of 18.48% *versus* 8.59% for neat PVDF. The membrane also exhibited excellent lithium electrode compatibility, maintaining stability over 16 days of storage.

The PVDF/PEO blend based SPE has been investigated by Dhaparwal and Sengwa,^158^ who investigated PVDF/PEO/LiCF_3_SO_3_ blend SPEs which clearly revealed that ionic transport in polymer blend is closely linked to crystal phases and polymer chain dynamics. The 75PVDF/25PEO blend matrix with 30 wt% salt achieved an unprecedented enhancement in ionic conductivity, exceeding 10^5^-fold improvement compared to neat PVDF. Additionally, increasing the PEO content from 25 wt% to 90 wt% the conductivity enhanced approximately by two orders of magnitude. A strong correlation between ionic conductivity and relaxation time was found suggesting that ion transport in these SPEs is closely associated with the segmental motion of polymer chains. Another study on PVDF/PEO demonstrated that preventing PEO crystallization within PVDF's crystalline regions enhanced amorphous phase content that contributes to the enhanced ionic conductivity of the SPE.^159,160^

The manipulation of polymorphism and crystalline phases in PVDF (will be discussed in detail in the last section of this article) are also critical in optimizing the electrochemical performance of PVDF-based membranes. For instance, PVDF/PMMA blends prepared *via* emulsion polymerization of MMA in the presence of PVDF latex seeds exhibited nanoscale β/γ phases due to nanoscale confinement (full PMMA coverage over PVDF resulted in nanoscale confinement), which enhanced dielectric properties and minimized hysteresis losses, supporting their use in high-performance energy storage systems.^161^ Innovative approaches, such as blending semi-interpenetrating polymer networks (semi-IPN) has also been explored.^152^ PVDF/PBA (poly(butyl acrylate)) blends with a semi-IPN structure was found to effectively prevent electrolyte leakage, achieving high electrolyte uptake (120%) and conductivity (0.81 mS cm^−1^ at room temperature). These membranes demonstrated excellent cycling stability, making them suitable for energy storage applications.

**Fig. 13:**
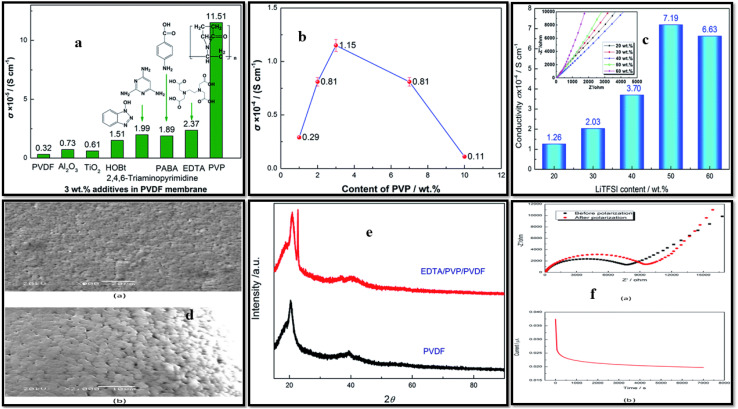
(a) Conductivity of different additives added to the PVDF, (b) effect of PVP content on conductivity, (c) effect of various compositions of LiTFSI salt on the EDTA/PVP/PVDF membrane conductivity, (d) SEM micrographs at 900× (a) and 2000× (b) of EDTA/PVDF/PVP membrane, (e) XRD profiles of pristine PVDF and membrane, (f) EIS before and after polarization (a) and *current vs. time* plot during the polarization process (b).^162^

Wang *et al.*^162^ explored the effects of electron-donating additives in PVDF on ionic conductivity. These additives include metal oxides (Al_2_O_3_ and TiO_2_), organic species such as ethylene diamine tetra acetic acid (EDTA), and polyvinylpyrrolidone (PVP) in PVDF matrices (Fig. 13). By comparing the ionic conductivity of the prepared compositions with 3 wt% additive, the highest conductivity of 1.15 × 10^−4^ S cm^−1^ was observed with PVP blend. In a separate study, blending EDTA and PVP with PVDF further reduced crystallinity (from 58.14% to 55.39%) that resulted in enhanced lithium-ion motion, yielding a conductivity of 7.17 × 10^−4^ S cm^−1^. Karabelli *et al.*^142^ fabricated crosslinked PVDF membranes using gamma radiation with crosslinking agents (TAIC (triallyl isocyanurate) and MEP (macromonomer of ethylene oxide-propylene oxide)). These crosslinked blend membranes achieved higher conductivity of 10 mS cm^−1^, reduced resistivity, and excellent mechanical stability, outperforming commercial cellulose membranes. PVDF/PAN blend membranes using two different techniques, namely thermally induced phase separation^144^ and electrospinning,^131^ have been found to improve mechanical and thermal stability and electrochemical performance. With 90 : 10 PVDF/PAN composition, the blend showed superior discharge stability and high C-rate performance compared to neat PVDF or Celgard® 2400 separators. PVDF/PAN nanofibrous membranes exhibited remarkable dimensional stability at elevated temperatures. Doping PAN into PVDF improved inter-fiber linkages, significantly enhancing mechanical strength, ionic conductivity, and electrolyte uptake.

## 2. Dielectric engineering in PVDF-based SPEs for enhanced electrochemical performance

The most distinctive electrical property of dielectric materials, when placed in external electric field, is their ability to become polarized, which fundamentally involves the redistribution and reorientation of electrical charges within the dielectric material. The net effect of these microscopic rearrangements is the generation of macroscopic polarization, which enables the material ability to store and manage electrical energy. Dielectric materials exhibit various polarization such as electronic, ionic, dipolar, thermal relaxation, and space-charge polarization. Each polarization operates within distinct frequency ranges and contributes differently to the overall polarization behavior of the material.^163,164^ The dielectric constant *ε*_r_, a macroscopic parameter, characterizes the degree of polarization induced within dielectric materials in response to an externally applied electric field.

Unlike liquid electrolytes, which attain high ionic conductivity through the use of high dielectric constant and low-viscosity solvents that promote lithium salt dissociation and ion mobility, solid electrolytes typically exhibit lower ionic conductivity owing to their limited capacity for lithium salt dissociation and ion transport.^165,166^ However, manipulating dielectric properties of the SPEs, can lead to excellent ionic conductivity and outstanding battery performance.^167–169^ In CPEs, the dielectric behavior assumes paramount importance, as a high dielectric constant of the medium facilitates lithium salt dissociation, thereby generating a greater abundance of free charge carriers and reducing the activation energy required for ion transportation. The optimization of the dielectric behavior of the PVDF-based SPEs can be achieved through the judicious design by selecting an appropriate polymorph of PVDF or its copolymers and or/dielectric fillers.^170,171^

**Fig. 14:**
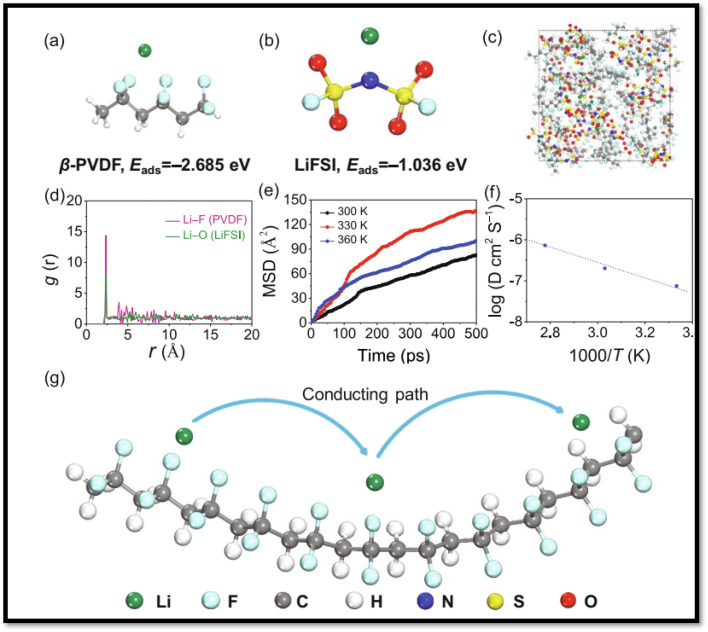
Optimized molecular structures and binding energy calculations for Li^+^ interactions with: (a) PVDF polymer matrix and (b) lithium bis(fluorosulfonyl)imide (LiFSI) salt. Dynamic analysis reveals: (c) representative atomic configuration from AIMD simulations of PVDF-LiFSI at 300 K, (d) radial distribution functions characterizing Li^+^ coordination environments: *g*(*r*) for Li–F (PVDF) and *g*(*r*) for Li–O (LiFSI). Transport properties: (e) temperature-dependent mean squared displacement of Li^+^ (300–360 K), (f) derived diffusion coefficients for Li^+^ migration, (g) visualized lithium-ion conduction pathways in PVDF-LiFSI solid polymer electrolyte. (Reproduced with permission from ref. 30).

**Fig. 15:**
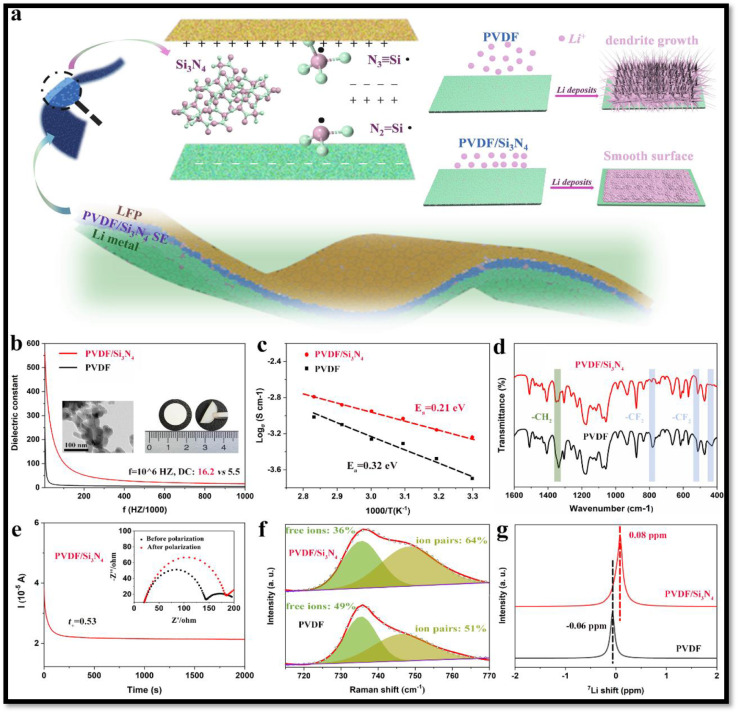
(a) Schematic representation of lithium deposition in the LFP cell constructed with PVDF and PVDF/Si_3_N_4_ electrolytes. (b) Presents the dielectric constants of PVDF and PVDF/Si_3_N_4_ membranes, along with TEM images of amorphous Si_3_N_4_ particles and a digital images of the flexible PVDF/Si_3_N_4_ membrane. Arrhenius plots illustrating the temperature-dependent behavior of PVDF and PVDF/Si_3_N_4_ electrolytes are shown in (c), while (d) displays the FT-IR spectra of these membranes. The current *vs.* time profile of the symmetric Li/PVDF/Si_3_N_4_ Li/cell incorporating the electrolyte is depicted in (e). Additionally, (f) presents the fitted Raman spectra, and (g) shows the solid-state ^7^Li-NMR spectra of PVDF and PVDF/Si_3_N_4_ membranes. Reproduced with permission from ref. 32.

Kang *et al.*^30^ developed a highly polar all-trans β-PVDF-based SPE using LiFSI as the lithium salt, which demonstrated an exceptionally large dielectric constant reaching 10^8^, significantly surpassing the 31.7 value of pure PVDF at 0.1 Hz. This enhancement was attributed to the increased dipole moment resulting from the separation of FSI^−^ and Li^+^ (salt polarization) due to the ion–dipole interactions between the aligned F atoms of the β-phase PVDF and the lithium ions. The highly polar all-trans β-PVDF, thus contributes to salt dissociation through its pronounced ‘solvating’ capability, enabling the SPE to achieve a high dielectric constant and remarkable ionic conductivity of 0.77 × 10^−3^ S cm^−1^. It was proposed that the unique structure of β-PVDF directs lithium cations to align along the PVDF chains, generating a unique pathway for lithium-ion hopping within the SPE. Additionally, first-principles simulations conducted by the authors further supported the proposed ion transport mechanism in the SPE, suggesting that lithium-ion movement is governed by ion–dipole interactions schematically depicted in Fig. 14. The assembled all-solid-state LiFePO_4_ battery using the PVDF–LiFSI SPE achieved a real capacity of up to 1.69 mA h cm^−2^ and demonstrated an exceptional cycling life exceeding 2600 h. Cheng *et al.*^32^ induced high dielectric constant in PVDF-based SPEs by the incorporating amorphous silicon nitride (Si_3_N_4_). The PVDF/Si_3_N_4_ CPE exhibited a reasonable ionic conductivity of 5.7 × 10^−4^ S cm^−1^ at room temperature, emphasizing the beneficial role of enhanced dielectric constant in facilitating charge transport. This improvement was further evidenced by a significant reduction in *E*_a_ from 0.32 eV to 0.21 eV, indicating lower ion transport barriers in the CPEs (Fig. 15). The high dielectric constant amorphous Si_3_N_4_ was observed to effectively suppress anion migration, screen external electric fields, enhance the Li^+^ transference number (*t*_+_ = 0.53 at 298 K), and inhibit dendrite growth during cycling. The developed CPE exhibited exceptional cycling stability, maintaining consistent performance for >250 h in symmetric Li|PVDF/Si_3_N_4_|Li cells at an elevated current density of 1.0 mA cm^−2^.

**Fig. 16:**
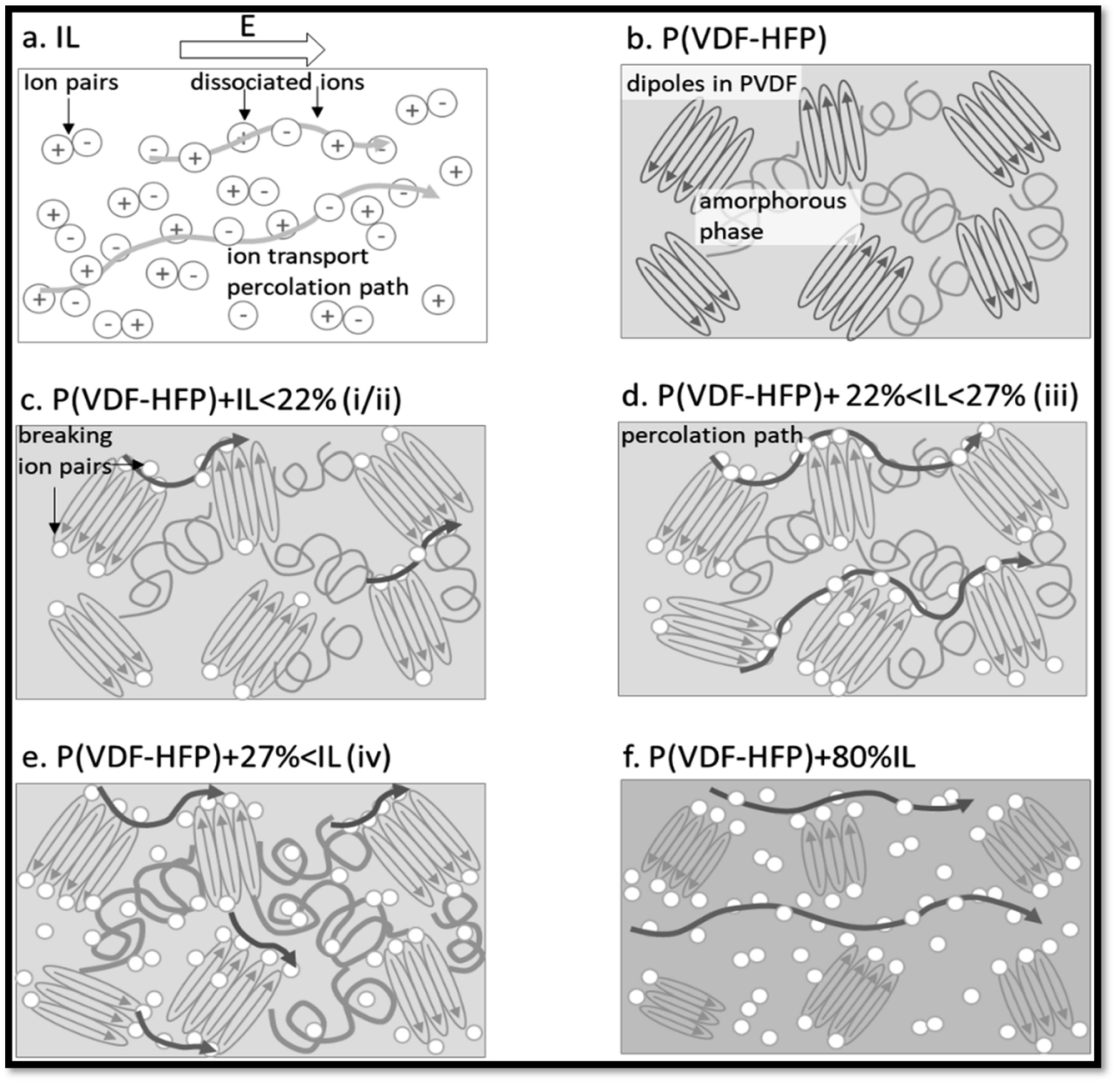
Illustrates the underlying mechanism of ion transport within the ferroelectric polymer. (a) In IL, dissociated ions form conduction channels between ion pairs. During conductivity measurements, *E* represents the direction of the applied electric field. (b) In P(VDF-HFP) matrix the amorphous HFP domains serve as the loading site for IL. (c) The introduction of small amount of IL induces the ion pairs dissociation *via* ion-PVDF dipole interactions. (d) As the IL concentration increases, conductivity reaches its peak, facilitated by the formation of a percolation channel at the interface between the crystalline and amorphous phases. (e) As the IL concentration continues to increase, the amorphous phase swells, disrupting the interconnected percolation pathways at the interface, leading to a decline in conductivity. (f) When a large amount of IL is introduced, the amorphous phase becomes fully saturated with ions, resulting in the formation of a percolation pathway like that observed in pure ionic liquids.

Electrospinning techniques can also be employed to promote dipoles polarization in SPEs. As an example, Sultana *et al.*^31^ induced the formation of highly polar all-trans β-phase (ferroelectric phase) PVDF in PVDF/(1-ethyl-3-methylimidazolium bis(trifuoromethylsulfonyl)imide (EMIM TFSI) as ionic liquid))-based SPE fiber mats through electrospinning. The authors demonstrated that the electro spun fiber has higher β-phase content compared to the corresponding film electrolyte that leads to higher dipole orientation, improved piezoelectric character, and higher conductivity. The ionic conductivity increased up to two orders of magnitude with increasing the β content within the matrix. Fig. 16 depicts the proposed underlying mechanism for the enhanced ionic conductivity of the fiber mats compared to the electrolyte film achieved by solution casting method.

The enhancement of dielectric behavior of CPEs can also be achieved by the incorporation of dielectric fillers, such as TiO_2_,^172^ Al_2_O_3_,^173^ SiO_2_,^174^ SrBi_4_Ti_4_O_15_,^175^ and BaTiO_3_ (BTO).^176^ Dielectric nanoparticles can lower the activation energy by providing efficient ion conducting pathways in the polymer matrix. Moreover, dielectric particles can bind anions and promote Li^+^ migration by acting as Lewis's acids.^175^ However, when fillers are added beyond optimum concentration, the agglomeration of nanoparticles decreases ionic conductivity and restricts carrier mobility.^176,177^ Shi *et al.*^178^ developed PVDF-based CPE by coupling dielectric BTO and conductive LLTO (BaTiO_3_–Li_0.33_La_0.56_TiO_3–*x*_) nanowires. BTO is a well-known perovskite ceramic dielectric with a high dielectric constant (*ε*_r_ ∼ 10^3^), which is primarily attributed to its ferroelectric properties. BTO, being ferroelectric, undergoes spontaneous polarization and when an external electric field is applied, it generates a polarization field that is opposite in direction of the external field. This reverse electric field weakens the space charge layer and reduces the Li^+^ concentration gradient that results in more salt dissociation generating more free charge carriers.

**Fig. 17:**
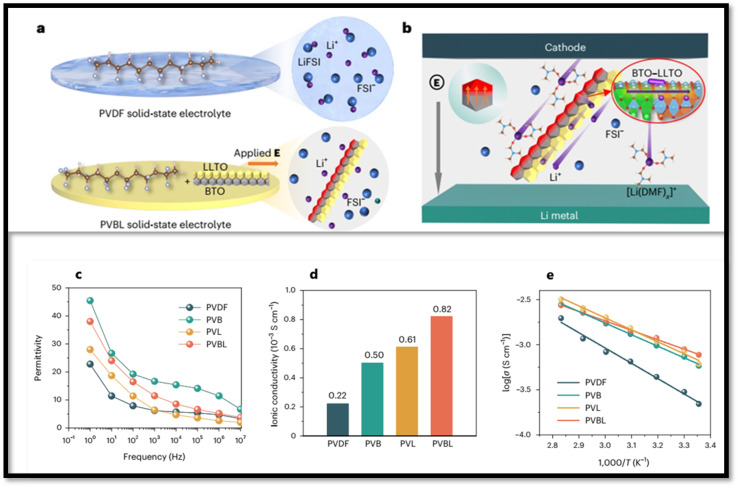
(a) Schematic comparison of Li-salt states in PVDF and PVBL electrolytes. (b) Proposed mechanism of improved salt dissociation and Li^+^ migration enabled by BTO-LLTO coupling in PVBL electrolyte. (c) Dielectric constants (*ε*_r_) and (d) ionic conductivities (*σ*) of PVDF-based electrolytes at 25 °C. (e) Arrhenius plots showing temperature-dependent ionic conductivity behavior. Nomenclature: PVB (PVDF with 15 wt% BTO nanowires), PVL (PVDF with 15 wt% LLTO nanowires), and PVBL (PVDF with 15% BTO–LLTO). Reproduced with permission from ref. 178.

The coupled BTO–LLTO nanowire structure, in addition to promoting salt dissociation, also weakens the space charge layer at the interfaces (due to polarization of the dielectric BTO) that enhances the transport efficiency of the dissociated Li^+^ in the CPE (Fig. 17). At 10 Hz and 25 °C, the relative dielectric constant, *ε*_r_ values of the electrolytes follow the order: PVDF (*ε*_r_ = 11) < PVL (*ε*_r_ = 18) < PVBL (*ε*_r_ = 24) < PVB (*ε*_r_ = 27) (Fig. 17c). The incorporation of BTO significantly enhances *ε*_r_ in all systems. Notably, PVBL, as shown in Fig. 17d exhibited the highest room temperature ionic conductivity (*σ*) (8.2 × 10^−4^ S cm^−1^), surpassing PVDF (2.2 × 10^−4^ S cm^−1^), PVL (6.1 × 10^−4^ S cm^−1^), and PVB (5.0 × 10^−4^ S cm^−1^). Furthermore, Arrhenius analysis reveals that the activation energy (*E*_a_ for ion migration in PVBL decreases from 0.34 eV (pristine PVDF) to 0.20 eV with BTO–LLTO addition (Fig. 17e, indicating improved ion mobility. Nomenclature in Fig. 17: PVB (PVDF with 15 wt% BTO nanowires), PVL (PVDF with 15 wt% LLTO nanowires), and PVBL (PVDF with 15% BTO–LLTO).

BTO is a ferroelectric ceramic without ion-conductive ability and hence it indirectly affects the lithium-ion conductivity in CPEs as discussed above. In contrast, a ferroelectric ceramic with ion conducting ability, such as LiTaO_3_ (LTO), could induce not only smooth bulk conductivity by diminishing the space charge layer and salt dissociation but also offer efficient Li^+^ transport pathways for enhanced ionic conductivity. As an example, Yuan *et al.*^179^ incorporated LTO filler in PVDF-based CPEs. LTO, which spontaneously polarizes under an applied electric field, weakens the space charge layer at the PVDF/filler interface to boost the Li^+^ transport. Further, being an ion conductor, LTO also supplies abundant efficient ion transport channels. The highest conductivity of 4.90 × 10^−4^ S cm^−1^ and cation transference number *t*_+_ = 0.45 were achieved by the resulting CPE. LTO generates a uniform electric field that facilitates uniform Li plating/stripping, enabling the Li/PVDF-LTO SPE/Li symmetric batteries to achieve superior cycling performance for 4000 hours at 0.1 mA cm^−2^ and 1000 hours at 0.5 mA cm^−2^ at room temperature. In addition, the high-voltage solid-state NCM811 (LiNi_0.8_Co_0.1_Mn_0.1_O_2_)/PVDF-LTO SPE/Li full batteries delivered excellent long cycling for 1400 cycles with capacity retention of 70% at 1C and endure 700 cycles at 2C.

**Fig. 18:**
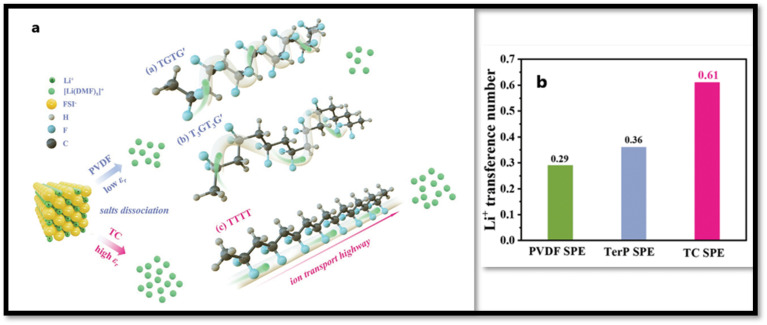
(a) Schematic presentation of salt dissociation and ion transport mechanisms enabled by solid polymer electrolyte (SPE) matrices with varying dielectric constants and polymer conformations, (b) lithium ion transference number of all the three SPEs measured at 25 °C. Reproduced with permission from ref. 187.

Despite the extensive characterization of the structural and dielectric behavior of PVDF polymorphic phases, it is often beneficial to incorporate its piezoelectric or ferroelectric homologues as comonomers to further enhance its dielectric polarization capabilities.^31,180,181^ For example, incorporation of tetrafluoroethylene (TrFE) as comonomer in PVDF stabilizes the all-trans conformation (β-phase), even at a relatively small TrFF ratio. Some of the comonomers, such as chlorotrifluoroethylene (CTFE) and hexafluoropropylene (HFP), which are larger in size than the VDF monomer, when copolymerized, results in the formation of gauche conformation that destabilizes the ferroelectric phase in the copolymers.^182,183^ The incorporation of CTFE, or chlorodifluoroethylene (CDFE), as third monomer in P(VDF-TrFE)-based copolymers introduces disorder into the polymer matrix. The random incorporation of these comonomers creates defects and reduces the size of ferroelectric domains, promoting the formation of nano-sized polar domains, a hallmark of relaxer ferroelectrics. These properties render the modified copolymers highly suitable for energy storage systems, owing to their elevated dielectric constant and minimal hysteresis losses.^184,185^ As an example, Liu *et al.*^186^ developed P(VDF-TrFE)/Li_6_PS_5_Cl electro spun SPE membranes, where the filler form Li^+^ ion conduction channels and the P(VDF-TrFE) offers flexibility to the electrolyte membrane. The authors showed that the strong polar interactions between the filler and the highly polar matrix contribute to the exceptional room temperature ionic conductivity of approximately 1.2 mS cm^−1^, along with the mechanical ductility of the CPE membrane. The fabricated all solid-state cells offered exceptional life cycle retaining 71% capacity after 20 000 cycles at 1.0 mA cm^−2^ (*i.e.*, 1.61C). Huang *et al.*^187^ designed a novel SPE matrix, with enhanced salt dissociation and ion transport capabilities by blending high-dielectric P(VDF-TrFE-CTFE) with all-trans P(VDF-TrFE). The authors argued that the all-trans P(VDF-TrFE) forces P(VDF-TrFE-CTFE) to orient in all-trans conformation from the mixed TGTG′ and T_3_GT_3_G′ orientations. With these all-F atoms located on one side of the chain form ion hopping channels as depicted in Fig. 18a. Also, the dielectric constant increased from ≈10 for PVDF to ≈33 for SPE matrix that facilitates salt dissociation. Thus, the SPE exhibited an increased conductivity of 2.37 × 10^−4^ S cm^−1^ and a high cation transference number, *t*^*+*^ = 0.61 (*vs.* 0.29 for PVDF SPE and 0.36 for Terpolymer SPE) at 25 °C as shown in Fig. 18b.

**Fig. 19:**
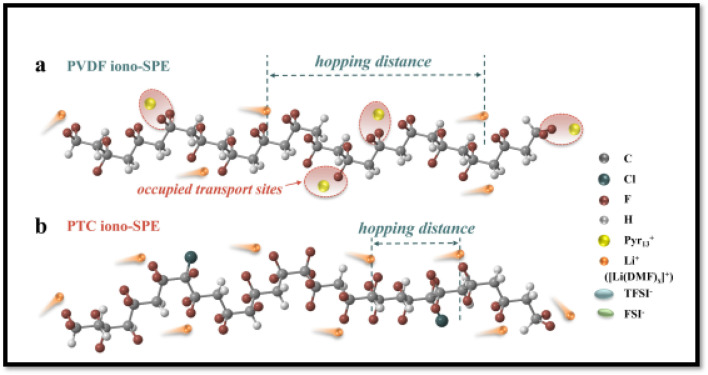
Schematic illustration of Li^+^ transport mechanism in (a) PVDF-iono SPE *versus* (b) PTC-iono SPE. Key observations: In the PVDF system (a), the occupation of transport sites by Pyr_13_^+^ cations increase the effective hopping distance for Li^+^ migration. The PTC system (b) demonstrates more efficient Li^+^ conduction pathways along polymer chains. Reproduced with permission from ref. 190.

**Fig. 20:**
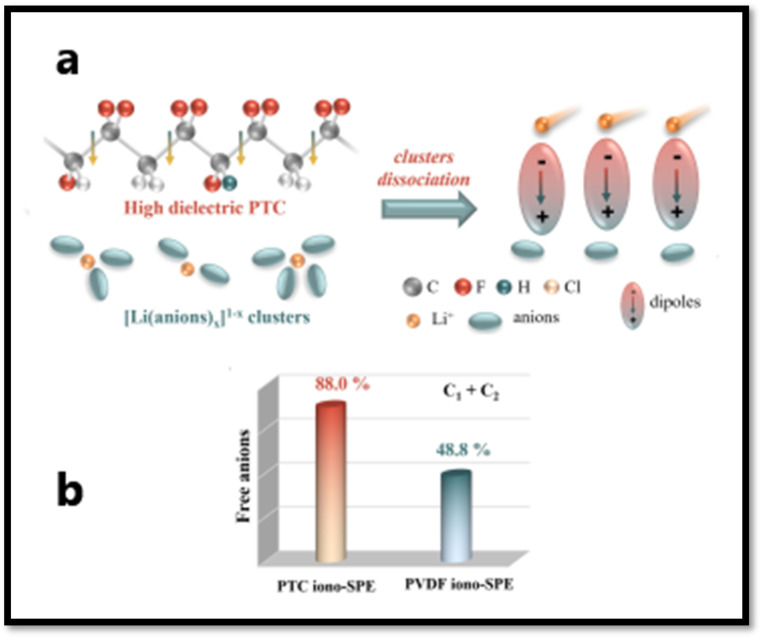
(a) Schematic illustration of [Li(anion)_*x*_]^1−*x*^ cluster dissociation facilitated by high-dielectric-constant PTC. (b) Comparative ratio of free anions in PTC-iono-based SPE and PVDF-iono-based SPE, as quantified by Raman spectroscopy analysis. Reproduced with permission from ref. 190.

Ionic liquid generally acts as plasticizer and improves segmental mobility of the polymer chains in SPEs, however, its free organic cations could occupy some transport sites for lithium ions by coordinating with electronegative segments of polymer chain.^188,189^ This might result in an increased hopping distance for lithium ions, which translates into a bigger energy barrier for lithium-ion transport. Also, the interaction between anions from ILs and lithium ions could negatively impact on the transport efficiency of lithium ions in polymer matrix. To address this challenge, Liu *et al.*^190^ developed a novel strategy for reducing the interaction of IL organic cation and the polymer matrix. The strategy involves reducing the binding energy between polymer chains and the added organic cations of IL (this facilitates easier movement of lithium ions within the polymer matrix) and boosting the dissociation of Li^+^-anion clusters (enhancing the availability of free lithium ions for conduction, improving overall ionic conductivity). In their work, they designed a highly dielectric P(VDF-TrFE-CTFE) (PTC) terpolymer with appropriate polarity as polymer matrix and developed SPE by incorporating Pyr_13_TFSI (ionic liquid, IL) and LiFSI as the lithium salt to prepare iono-SPE. The PTC with its moderate polarity exhibits lower adsorption energy of 0.20 eV *vs.* 0.81 eV for PVDF with Pyr_13_^+^, which makes the interaction of organic cation and PTC less favorable and hence minimizes the chances of organic cation to occupy the lithium ion hopping sites on polymer chains as schematically shown in Fig. 19. As result, the energy barrier for ion Li^+^ transport reduces from 0.35 eV for PVDF-based iono-SPE to 0.25 eV for PTC-based iono-SPE. Further, PTC, as depicted in Fig. 20, with its high dielectric constant (∼40.2) compared to PVDF (∼11.7) increases the free Li^+^ ion concentration by inducing the Li^+^-anion cluster dissociation that also contributes to enhanced ionic conductivity of PTC iono-SPE of 5.75 × 10^−4^ S cm^−1^ at 25 °C. These two effects also contribute to the suppression of lithium dendrites growth by maintaining a uniform Li^+^ flux. With this design, the LiFePO_4_/PTC iono-SPE/Li cells retained 91.5% cell capacity after 1000 cycles at 1C and 25 °C.

## 3. Machine learning for PVDF-based composite electrolyte design

The growing accumulation of experimental and computational data has propelled materials science into the era of machine learning (ML) and big data analytics, enabling the construction of predictive models and interpretation of large-scale datasets.^191^ These tools have revolutionized innovation across disciplines such as medical research, life sciences, and chemistry, giving rise to interdisciplinary fields like cheminformatics, medical informatics, and bioinformatics.^192–194^ For instance, data-driven approaches have been employed to simulate, design, and screen novel therapeutic compounds tailored to specific medical conditions.^195,196^

In materials science, ML has emerged as a powerful complement to experiments and simulations, accelerating the discovery of diverse materials ranging from LIBs^197^ to polymers for energy applications.^198^ By providing inexpensive and accurate property predictions, ML models help guide experimental efforts toward materials that meet target design criteria.^199^ Although the complexity of polymer systems presents challenges, recent studies have successfully applied ML to advance polymer separation membranes,^200^ polymer solar cells,^201^ and polymer dielectrics.^202^

The analysis of large datasets and advanced ML algorithms holds immense potential to expedite the discovery, characterization, and optimization of energy storage materials,^203–205^ while reducing reliance on trial-and-error experimentation.^206^ Most polymer electrolytes are loaded with plasticizers/nanofiller to achieve enhanced ionic conductivity and battery performance that results in significant complexity rendering these systems computationally challenging.^207–209^ Nevertheless, a number of ML-based studies have been successfully conducted on SPEs for LIB applications.^207,210–212^

**Fig. 21:**
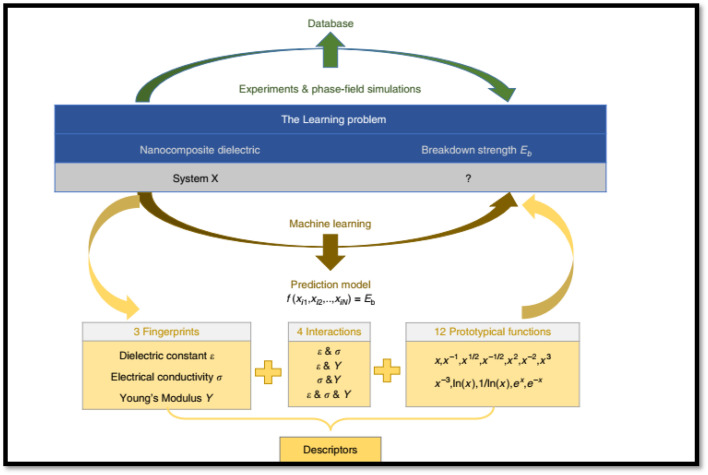
The schematic workflow illustrates ML approach for deriving an analytical expression to predict the breakdown strength of PVDF–HFP nanocomposites. This strategy uses a database breakdown strength derived from high-throughput phase-field simulations. Reproduced with permission from ref. 214.

ML-guided filler design can offer a fast track pathway to accelerate the discovery and optimization of composites for energy storage application by predicting the complex relationships between filler properties and polymer morphology.^200,212–214^ With relevant to the application of ML tools to PVDF-based composites only a limited number of studies have been carried out. For instance, Shen *et al.*^214^ developed an electrical–thermal–mechanical phase-field model to elucidate the dielectric breakdown mechanisms in PVDF-HFP-based nanocomposites. The developed ML strategy is schematically depicted in Fig. 21. The model uncovers a temperature-dependent transition in the breakdown behavior of PVDF-HFP: from electrically dominated breakdown at low temperatures to electrothermal breakdown at intermediate temperatures, and finally to coupled electrical–thermal–mechanical breakdown at elevated temperatures. By systematically analyzing dielectric constants, electrical conductivity, and Young's modules and the contributions of electric field energy, Joule heating, and strain energy, the authors established a general principle to classify breakdown mechanisms across diverse composite dielectrics. To extend these insights to nanocomposites, high-throughput phase-field simulations were employed to construct a dataset correlating nanofiller (various nanofillers were employed Al_2_O_3_, SiO_2_, MgO, and TiO_2_) properties (dielectric constant, conductivity, Young's modulus) with breakdown strength in PVDF-HFP-based composites. ML was then applied to derive an analytical expression for predicting breakdown strength as a function of these parameters. This expression enables rapid screening of nanofillers, consistently predicting enhanced breakdown strength compared to the pure PVDF, a trend validated by both simulations and experiments. The authors argued that the developed framework is generalizable to other nanofiller morphologies (*e.g.*, nanofibers, nanosheets, arbitrary geometries) and provides a theoretical strategy for optimizing polymer nanocomposite dielectrics. By identifying nanofillers that maximize breakdown performance, this work bridges computational design and experimental synthesis, offering actionable guidance for developing high-energy-density materials and devices. In a another study by Shen *et al.*^213^ on PVDF-BaTiO_3_-based nanocomposites, they constructed a continuum phase-field model to study electrostatic breakdown propagation. The model enables high-throughput computational screening of microstructure effects on dielectric constant, breakdown strength, and energy density. The results revealed that the breakdown pathways and strength are highly sensitive to the shape and orientation of nanofillers. The model predictions were found to align well with experimental data, validating its predictive capability. Based on the outcome, they performed high-throughput calculations to identify microstructures with optimal energy density, which led them to design and optimize an artificial sandwich microstructure, which achieved 2.44 times increase in energy density in PVDF–BaTiO_3_ nanocomposites compared to pure PVDF.

**Fig. 22:**
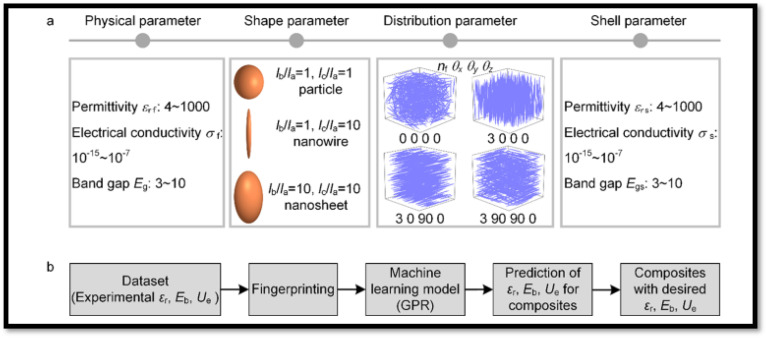
ML workflow. (a) Schematic fingerprint representation of composites with various schemes of fillers doping. (b) Schematic of ML workflow for predicting *ε*_r_, *E*_b_, and *U*_e_. Reproduced with permission from ref. 198.

Zhu *et al.*^198^ developed an ML model based on Gaussian Process Regression to rationally design composites with targeted dielectric constant (*ε*_r_), dielectric break down strength (*E*_b_), and discharge energy density (*U*_e_) using the available existing measured data in literature. The data set includes various polymers, such as PVDF, PMMA, *etc.* and various nanofillers such as Al_2_O_3_, TiO_2_, and BaTiO_3_. The ML workflow is schematically depicted in Fig. 22. The developed ML model assessed how nanofiller physical parameters, interface properties with the matrix, and geometric microstructure influence key dielectric properties (*ε*_r_, *E*_b_, *U*_e_). Analysis showed an inverse correlation: nanofiller dielectric constant and bandgap impact dielectric strength (*E*_b_) and dielectric constant (*ε*_r_) in opposing ways, making simultaneous improvement *via* nanofiller selection challenging. However, the *E*_b_ − *ε*_r_ trade-off can be managed by engineering nanofiller shape, orientation, and distribution. For both high-dielectric constant (*e.g.*, BaTiO_3_) and wide-bandgap fillers (*e.g.*, Al_2_O_3_), horizontally aligned nanosheets or orthotropic nanowires maintain or enhance the polymer's inherent breakdown strength, enabling high *U*_e_. Alternatively, vertically aligning high-dielectric constant fillers significantly boosts *ε*_r_, also yielding impressive *U*_e._

**Fig. 23:**
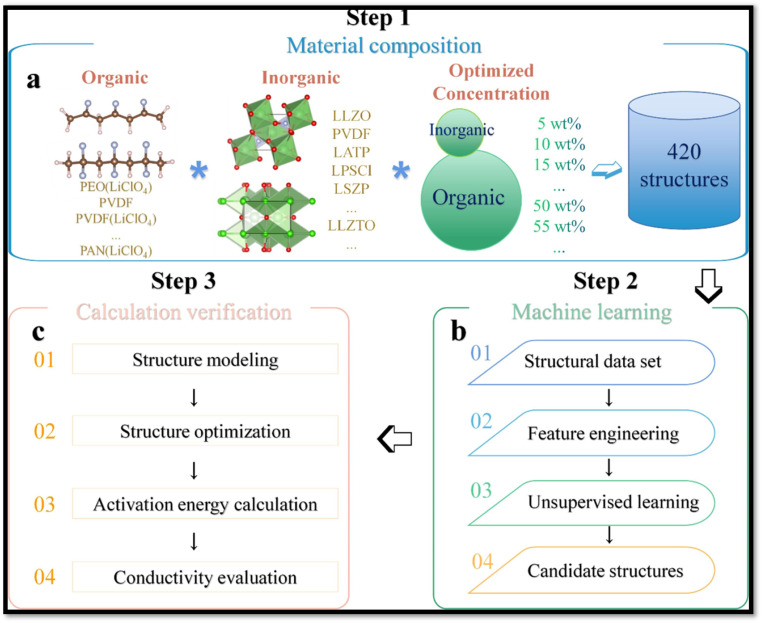
Illustration of the SCE discovery workflow: (a) compilation and preprocessing of 420 solid composite electrolyte (SCE) structures; (b) identification of promising candidates through an unsupervised learning approach; and (c) precise validation of the selected candidates using density functional theory (DFT) calculations. The arrows indicate the step-by-step progression of the methodology. Reproduced with permission from ref. 215.

Ina typical example with PVDF-based CPEs, Tao *et al.*^215^ introduced a novel unsupervised learning (UL) framework to accelerate the discovery of CPEs with active filler which are critical for enhancing safety and electrochemical stability (Fig. 23). Traditional experimental and high-throughput computational techniques are often constrained by time and data scarcity, posing challenges for rapid materials innovation. To address these limitations, the authors proposed Low-dimensional component (LDC) vector descriptor derived from elemental properties and ideal concentrations, which effectively captures compositional features of CPEs [combining inorganic nanofillers (*e.g.*, LLTO, LLZO) with polymer matrices (PEO, PVDF, PAN) and lithium salts (LiClO_4_, LiTFSI)] and is transferable to other materials domains. Through this approach, the screening space was significantly reduced, from 420 to just 49 candidate structures, resulting in a computational time saving equivalent to approximately 23 years on a 24-CPU supercomputing platform. Notably, five representative CPEs (LLTO-PEO (LiClO_4_), LLTO-PVDF (LiTFSI), LLZO-PAN (LiClO_4_), LLZO-PVDF (LiClO_4_), and LLZO-PVDF (LiTFSI)) were experimentally validated for ionic conductivity (*σ*), establishing the efficacy of the model. The unsupervised learning (UL) framework was primarily composed of three components: training algorithms, feature engineering, and dataset construction. Fig. 23 Illustrates the steps of the SCE discovery workflow. The process began with assembling a dataset by analyzing 45 groups of known data, from which 15 polymers (or lithium salts) and 14 active inorganic fillers (AIFs) were identified. High and low concentration levels were determined based on the median values of known optimal compositions. This led to the formation of a dataset comprising 420 distinct candidate SCE structures for UL-based clustering.

We have not found any reasonable number of reports in literature on the application of ML tools in the design of PVDF-based SPEs or CPEs,^210^ particularly on the ML-guided filler selection for PVDF-based CPEs. This section, however, particularly highlights pioneering studies where ML accelerated the design of ceramic/PVDF nanocomposite dielectrics, providing templates for PVDF-based SPEs and CPEs. The findings and outcomes of these pioneering studies will be instrumental in the application of ML tools in predicting and tailoring design PVDF-based CPEs for achieving the desired ionic conductivity and battery performance.^194,214^ We believe this section will offer a new perspective on ML-assisted design of PVDF-based SPEs and CPEs as efficient and robust energy storage systems

## 4. Conclusions and future work

This review offers recent advancements in PVDF-based blend SPEs and CPEs for their applications in all solid-state lithium-ion batteries (ASSLIBs), with a particular focus on the various factors that influence their ionic conductivity, including crystallinity, glass transition temperature, surface morphology, electrochemical properties, ion transport mechanisms, and mechanical properties. The conventional LIBs employing liquid electrolytes, such as organic carbonates *etc.*, are plagued by safety concerns, including leakage, weak mechanical properties, and explosions. In contrast, SPEs offer several benefits, including ease of thin-film formation, design flexibility, high mechanical strength, and optimal electrolyte/electrode contact. PVDF has emerged as a promising polymer host for SPEs due to its exceptional film-making properties, good compatibility with electrodes, and superior mechanical properties. Nevertheless, the high crystalline behavior and low ionic conductivity of PVDF pose significant challenges. To overcome these hurdles and enhance the electrochemical performance of PVDF-based electrolytes, this review has highlighted various smart strategies particularly in PVDF-based composites, blends electrolytes, and dielectric engineering. Compositing PVDF with various fillers, including zeolites, ceramic oxides, and carbon nanotubes, has been shown to enhance battery performance by increasing thermal stability, mechanical strength, and ionic conductivity. Blending PVDF and other polymers (PEO or PMMA *etc.*) can modify crystallinity, optimize phase separation, and facilitate ion transport by reducing the degree of crystallization, thereby increasing amorphous regions for better ion mobility. Additionally, polymer blends can enhance thermal and electrochemical stability, ensuring long-term performance and safety in energy storage applications. A detailed and comprehensive account of various tailored strategies for manipulating the dielectric behavior of the PVDF based SPEs has been discussed, emphasizing the importance of dielectric properties, their role in salt dissociation, ion mobility, and prevention of lithium metal dendrite formation. In recent times, machine learning (ML) has become a valuable tool for accelerating the design and optimization of SPEs. By analyzing extensive data on polymer structures and properties, these techniques can predict promising PVDF-based materials with improved ionic conductivity and mechanical strength. Combining computational predictions with experiments can speed up development and reduce costs, offering a powerful approach to tailor SPEs for advanced battery applications. Despite significant progress in research, further innovations are needed to enhance processability, accessibility, and sustainability. The development of novel SPEs with acceptable electrochemical, thermal, and mechanical performance is crucial for the widespread adoption of ASSLIBs. In the future, researchers may employ strategies such as modifying the polymer host with functional groups to restrict crystallinity, designing lithium salts as plasticizers, and developing single-ion conducting SPEs (SIC-PEs) by incorporating layered double hydroxide (LDH) fillers. Systematic and continuous exploration of polymer and inorganic filler combinations with enhanced overall performance is crucial for further reducing interfacial impedance and addressing solid–solid contact challenges between electrodes and electrolytes. The materials genome database can be utilized for cost-effective performance analysis and fabrication of efficient SPEs. The design of PVDF-based copolymers, CPEs, and blends SPEs with fast Li^+^ transport networks require a thorough understanding of the ion conduction mechanism. Moreover, future research should focus on underexplored PVDF copolymers (PVDF-TrFE, PVDF-CTFE, PVDF-CTFE-HFP) and their combination with diverse dielectric nanofillers, that could result unique dielectric, electrochemical, and mechanical performance. Additionally, establishing clear mechanistic correlations between the dielectric behavior of the PVDF-based electrolytes and dendrite formation or suppression could unlock new pathways to improve safety and longevity in solid-state batteries, an area that remains critically important yet insufficiently addressed. Further, significant potential exists for exploiting machine learning (ML) algorithms and artificial intelligence tools to tailor the design of PVDF-based electrolytes through systematic exploration of the vast compositional spaces of PVDF, its copolymers, and composite formulations. By predicting key performance indicators such as filler selection, ionic conductivity, morphology, and dendrite suppression potential, ML tools can potentially guide experimental efforts more efficiently in the rational design of PVDF-based SPEs and PCEs with desired conductivity and electrochemical performance. Ultimately, the development of PVDF-based SPEs will not be confined to LIBs alone but will further expand to various electrochemical devices, such as Zn-based batteries, supercapacitors, Mg-based batteries, and alkali-metal ion batteries.

## Author contributions

KHK: conceptualization, writing-original draft; AH: help in writing some topics, editing, SA: writing a few topics; AS: concept and editing; HH: conceptualization, supervision, and revision.

## Conflicts of interest

The authors affirm that there are no conflicts of interest, financial or otherwise, related to this work.

## Data Availability

The data supporting this study will be provided upon reasonable request.
